# IDH2-mediated regulation of the biogenesis of the oxidative phosphorylation system

**DOI:** 10.1126/sciadv.abl8716

**Published:** 2022-05-11

**Authors:** Anjaneyulu Murari, Naga S. V. Goparaju, Shauna-Kay Rhooms, Kaniz F. B. Hossain, Felix G. Liang, Christian J. Garcia, Cindy Osei, Tong Liu, Hong Li, Richard N. Kitsis, Rajesh Patel, Edward Owusu-Ansah

**Affiliations:** 1 Department of Physiology and Cellular Biophysics, Columbia University Irving Medical Center, New York, NY 10032, USA.; 2 Department of Cell Biology, Albert Einstein College of Medicine, Bronx, NY 10461, USA.; 3 Wilf Family Cardiovascular Research Institute, Albert Einstein College of Medicine, Bronx, NY 10461, USA.; 4 Center for Advanced Proteomics Research, Department of Microbiology, Biochemistry and Molecular Genetics, Rutgers University—New Jersey Medical School, Newark, NJ 07103, USA.; 5 Department of Pathology and Laboratory Medicine, Robert Wood Johnson Medical School, Piscataway, NJ 08854, USA.; 6 The Robert N. Butler Columbia Aging Center, Columbia University Irving Medical Center, New York, NY 10032, USA.

## Abstract

Several subunits in the matrix domain of mitochondrial complex I (CI) have been posited to be redox sensors for CI, but how elevated levels of reactive oxygen species (ROS) impinge on CI assembly is unknown. We report that genetic disruption of the mitochondrial NADPH-generating enzyme, isocitrate dehydrogenase 2 (IDH2), in *Drosophila* flight muscles results in elevated ROS levels and impairment of assembly of the oxidative phosphorylation system (OXPHOS). Mechanistically, this begins with an inhibition of biosynthesis of the matrix domain of CI and progresses to involve multiple OXPHOS complexes. Despite activation of multiple compensatory mechanisms, including enhanced coenzyme Q biosynthesis and the mitochondrial unfolded protein response, ferroptotic cell death ensues. Disruption of enzymes that eliminate hydrogen peroxide, but not those that eliminate the superoxide radical, recapitulates the phenotype, thereby implicating hydrogen peroxide as the signaling molecule involved. Thus, IDH2 modulates the assembly of the matrix domain of CI and ultimately that of the entire OXPHOS.

## Teaser

Isocitrate dehydrogenase 2 regulates ferroptosis, the mitochondrial unfolded protein response, and OXPHOS assembly in *Drosophila*.

## INTRODUCTION

Mammalian CI (NADH: ubiquinone oxidoreductase) contains 44 distinct subunits, one of which is present as two copies, giving rise to a total of 45 subunits. Fourteen subunits conserved from the ancestral enzyme in prokaryotes to eukaryotes constitute the minimal form of the enzyme. Consequently, they are referred to as the core or central subunits, as they contain all the catalytic centers of the enzyme. The additional 30 unique subunits are referred to as accessory subunits. The 45 subunits are organized into two arms of the complex—referred to as the matrix and membrane domains—that are oriented almost orthogonally to each other, resulting in a boot-shaped structure (*1*). There are three distinct functional modules of CI referred to as the N-, Q-, and P-modules. The N-module is the site of NADH oxidation in the matrix domain and contains the flavin mononucleotide (FMN) cofactor. The Q-module connects the N-module to the membrane domain and functions as the conduit for electron transfer to ubiquinone. The proton-pumping P-module is localized to the membrane domain [reviewed in (*2*–*4*)].

Several high-resolution micrographs have defined the locations of the accessory subunits in atomic detail and have shown that some have cofactors or features that make them excellent candidates for sensing the redox status of the cell (*5*–*7*). For instance, the N-module subunit, NDUFS6, which interacts with Zn^2+^, has been proposed as a redox sensor. Similarly, a thioredoxin-like subunit, NDUFA2, also located in the N-module, has a pair of cysteine residues that could also serve as a redox sensor through disulfide bonding. As Fe-S clusters as a group are very sensitive to inhibition by oxidative stress, any or all of the eight Fe-S clusters in the matrix domain could, in principle, serve as redox sensors. Accordingly, we explored the link between redox homeostasis and CI assembly.

As a major source of reactive oxygen species (ROS), mitochondria are equipped with a sophisticated antioxidant system that effectively buffers the organelle from the deleterious effects of ROS. Mitochondria have co-opted moderate levels of ROS as signaling molecules that can be used to communicate to the nucleus to alter gene expression as part of an integrated stress response (ISR) (*8*, *9*). The FMN molecule in the N-module is a major ROS-generating site in CI (*10*, *11*). The initial ROS generated is the superoxide radical, which can be converted to hydrogen peroxide by the mitochondria-localized superoxide dismutase 2 (SOD2). There are also cytosolic and extracellular isoforms of SOD (i.e., SOD1 and SOD3, respectively).

Elimination of hydrogen peroxide represents a major step in ROS detoxification, as it prevents the formation of the highly reactive hydroxyl radical, which can be formed when hydrogen peroxide reacts with Fe^2+^ in the Fenton reaction. Hydrogen peroxide generated from the activity of the various SODs, as well as other enzymes such as xanthine oxidase, can be subsequently reduced to water by catalase or glutathione peroxidase. Glutathione peroxidase uses reduced glutathione (GSH) to eliminate hydrogen peroxide and lipid hydroperoxides, in a process that converts GSH to the oxidized form (GSSG). GSSG is recycled to GSH by glutathione reductase (GR) in a reaction that also converts NADPH to NADP^+^. For that reason, NADPH-generating enzymes in the mitochondrion are a critical component of the mitochondrial antioxidant system. Several peroxiredoxins and thioredoxins also contribute to the antioxidant buffering capacity of the mitochondrion.

Ferroptosis is a nonapoptotic form of cell death that is triggered by iron-dependent lipid peroxidation (*12*). Although ferroptosis has not been studied extensively in skeletal muscles, all the hallmarks of ferroptosis are present in damaged or aged muscles (*13*–*15*). An understanding of the signals that regulate ferroptosis in muscles may lead to therapies for common aging-related and degenerative sarcopenic disorders. Hence, we also explored a potential connection between disruption of isocitrate dehydrogenase 2 (IDH2), oxidative stress, and ferroptosis.

We previously delineated the mechanism of CI assembly in *Drosophila* flight (thoracic) muscles, demonstrating conservation from fly to mammalian systems (*2*, *16*, *17*). This baseline information, together with its classical genetics and copious mitochondria, makes the *Drosophila* flight muscles an ideal system in which to dissect the mechanistic link between ROS formation and oxidative phosphorylation (OXPHOS) system assembly in vivo. As a consequence, we manipulated the expression of several genes with antioxidant activity and examined their effects on OXPHOS assembly. We find that RNA interference (RNAi)–mediated disruption of enzymes that eliminate hydrogen peroxide, but not those that eliminate the superoxide radical, impairs the assembly of respiratory complexes. In particular, induction of ROS by genetic disruption of IDH2 impairs the assembly of CI. Mechanistically, this begins with an inhibition of biosynthesis of the Q- and N-modules in the matrix domain of CI, but eventually involves multiple OXPHOS complexes. Ferroptosis is induced, despite the induction of multiple compensatory responses. The same phenotype can be recapitulated by disruption of the mitochondrial NADP-dependent isoform of malate dehydrogenase and can be partially ameliorated by overexpression of an alternative NADH–ubiquinone oxidoreductase, NDI1. We propose a model in which mitochondrial NADPH-generating enzymes modulate OXPHOS assembly and that signals arising when assembly is disrupted activate ferroptosis in muscles.

## RESULTS

### RNAi-mediated disruption of the *Drosophila* ortholog of IDH2 (CG7176) and other antioxidant enzymes in *Drosophila* flight muscles impairs OXPHOS assembly

**Fig. 1.**
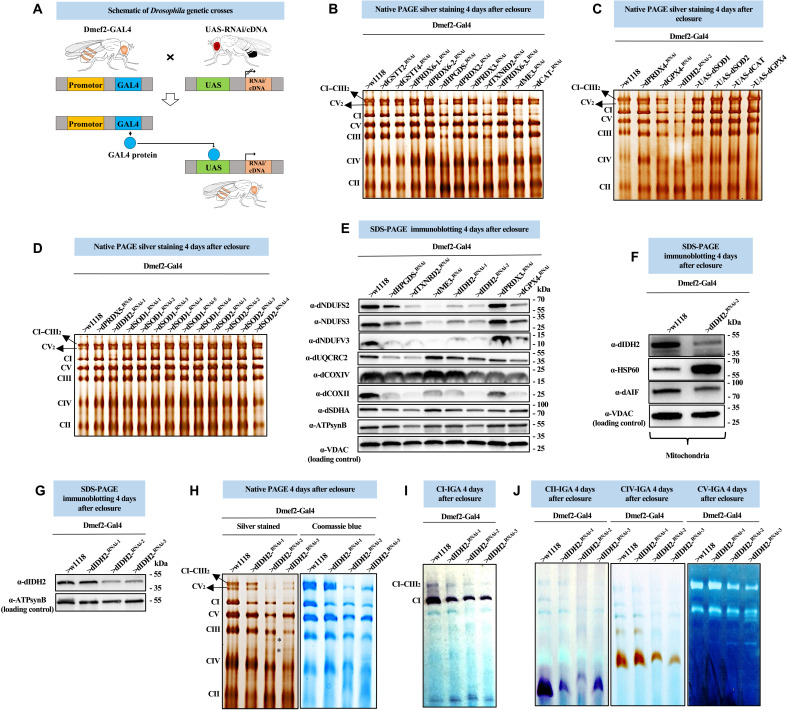
RNAi-mediated disruption of the *Drosophila* ortholog of IDH2 (CG7176) and other antioxidant enzymes in *Drosophila* flight muscles impairs OXPHOS assembly. (**A**) Schematic describing the Gal4-UAS system. (**B** to **D**) Mitochondrial preparations from adult flight muscles expressing the UAS-RNAi or UAS-cDNA constructs shown in muscles using Dmef2-Gal4 were analyzed by silver staining of native gels (see Materials and Methods). Samples expressing RNAi constructs for dME3 were analyzed 2 days after eclosure due to early lethality. For some of the dSOD1 and dSOD2 RNAi lines, lethality began by 3 days, but a few flies survived to 5 days. Hence, we dissected flies at 4 days. (**E**) Total tissue lysates from flight/thoracic muscles isolated from flies with the genotypes listed were analyzed by SDS–polyacrylamide gel electrophoresis (SDS-PAGE) and immunoblotting with anti-dNDUFS2, anti-NDUFS3 (which detects dNDUFS3), anti-dNDUFV3, anti-dUQCRC2, anti-dCOXIV, anti-dCOXII, and anti-dSDHA. (**F**) Mitochondrial preparations from thoraces of flies with the genotypes listed were analyzed by SDS-PAGE and immunoblotting with anti-dIDH2, anti-dAIF, and anti-Hsp60 (which detects dHsp60). (**G**) Total tissue lysates from flight/thoracic muscles from flies with the genotypes shown were analyzed by SDS-PAGE and immunoblotting with anti-dIDH2. (**H**) Mitochondrial preparations from thoraces of flies with the genotypes listed were analyzed by BN-PAGE followed by silver staining (left panel) and Coomassie blue staining (right panel). The asterisk (*) denotes accumulated assembly intermediates (AIs). (**I** and **J**) CI, CII, CIV, and CV in-gel activity (IGA) assays. See figs. S1 to S3 for replicates and quantification of immunoblots in (E) to (G). Loading controls used in the western blots are as indicated.

The flight muscles in *Drosophila* are highly enriched with mitochondria and have been used extensively in the past to study the functions of proteins that regulate mitochondrial function (*17*–*22*). Accordingly, to explore the possible role of ROS metabolism in OXPHOS assembly, we examined the effect of knocking down or overexpressing various antioxidant enzymes on the assembly of OXPHOS complexes in flight muscles using the Gal4/UAS system (*23*). Specifically, female flies carrying the Dmef2-Gal4 transgene were mated with various male flies, each of which expressed either a UAS-RNAi construct or a UAS-cDNA transgene of a particular antioxidant enzyme. This results in offspring that have a specific antioxidant enzyme knocked down or overexpressed, respectively, in flight muscles (Fig. 1A) (*24*).

We knocked down the expression of genes encoding for *Drosophila* orthologs of SOD1 (dSOD1/CG11793), SOD2 (dSOD2/CG8905), catalase (dCAT/CG6871), IDH2 (dIDH2/CG7176), the NADPH-dependent malate dehydrogenase (dME3/CG5889), multiple peroxiredoxins (dPRDX2/CG1633, dPRDX3/CG5826, dPRDX4/CG1274, dPRDX5/CG7217, dPRDX6-1/CG3083, dPRDX6-2/CG12405, and dPRDX6-3/CG11765), multiple glutathione *S*-transferases (dGSTT2/CG10045, dGSTT4/CG12242, and dHPGDS/CG8938), thioredoxin 1 (d*TXNRD2/CG2151*), and glutathione peroxidase 4 (dGPX4*/CG12013*) in flight muscles (see Materials and Methods). Subsequently, we isolated mitochondria from the flight muscles of adult flies expressing various RNAi constructs, solubilized their membranes in digitonin, and analyzed the integrity of their OXPHOS complexes using silver staining of blue native gels (Fig. 1, B to D). RNAi-mediated disruption of *dME3*, *dCAT*, and one of the RNAi constructs for *dIDH2* (UAS-dIDH2^-RNAi-1^) impaired CI assembly (Fig. 1, B and D). On the other hand, RNAi-mediated knockdown of *dHPGDS*, *dTXNRD2*, *dGPX4*, and a second transgenic RNAi construct targeting *dIDH2* (UAS-dIDH2^-RNAi-2^) impaired the assembly of multiple OXPHOS complexes, in particular, CI and complex III (CIII) (Fig. 1, B and C). Unexpectedly, none of the multiple transgenic RNAi lines targeting *dSOD1* and *dSOD2*—including a few that were potent enough to cause the flies to succumb to lethality beginning as early as 3 days after eclosing as adults—notably impaired OXPHOS assembly (Fig. 1D). In addition, none of the antioxidant enzymes we overexpressed (i.e., dSOD1, dSOD2, dCAT, and dGPX4) disrupted OXPHOS assembly (Fig. 1C). Immunoblotting of OXPHOS complexes from samples where the RNAi lines showed phenotypes confirmed the silver staining results (Fig. 1E and figs. S1 to S3). These results indicate that a failure to eliminate the superoxide radical, while deleterious, does not appear to alter OXPHOS assembly. In contrast, all the enzymes that produced an assembly defect when disrupted are required for eliminating hydrogen peroxide or lipid peroxides (either directly or indirectly).

IDHs catalyze the oxidative decarboxylation of isocitrate to 2-oxyglutarate and a reduced pyridine nucleotide cofactor. Three major isoforms of IDH can be distinguished on the basis of their subcellular localization and type of pyridine nucleotide cofactor used to catalyze their enzymatic reaction. IDH1 (localized to the cytosol and peroxisomes) and IDH2 (localized to the mitochondrial matrix) use NADP^+^ as a cofactor. IDH3, which is also localized to the mitochondrial matrix, uses NAD^+^ as a cofactor. CG7176 (dIDH2) is the sole *Drosophila* ortholog of both IDH1 and IDH2, suggesting that it could function in the cytosol, peroxisome, and/or mitochondrion. We used an antibody we raised against dIDH2 to confirm that at least a portion of dIDH2 localizes to the mitochondrion (Fig. 1F and figs. S1 to S3).

We knocked down dIDH2 expression in flight muscles using three different transgenic RNAi constructs and compared the integrity of their OXPHOS complexes using silver staining and blue native polyacrylamide gel electrophoresis (BN-PAGE). The genotypes of the flies were *Dmef2-Gal4/UAS-dIDH2^-RNAi-1^* (dIDH2-KD1), *Dmef2-Gal4/UAS-dIDH2^-RNAi-2^* (dIDH2-KD2), and *Dmef2-Gal4/UAS-dIDH2^-RNAi-3^* (dIDH2-KD3). Immunoblotting analyses revealed that the expression of dIDH2 had been significantly reduced in flight muscles obtained from dIDH2-KD2 and dIDH2-KD3 flies (Fig. 1G and figs. S1 to S3). In contrast to the specific CI assembly defect observed in dIDH2-KD1 flight muscles, the assembly of multiple complexes was impaired in mitochondria from dIDH2-KD2 and dIDH2-KD3 flies (Fig. 1H). In line with the silver staining and Coomassie staining results described in Fig. 1H, in-gel CI activity was reduced in all three genotypes (Fig. 1I). In-gel complex II (CII), complex IV (CIV), and complex V (CV) activities were also reduced in mitochondria from dIDH2-KD2 and dIDH2-KD3 flies (Fig. 1J). Together, these observations establish a progression of severity of phenotypes produced by the three transgenic RNAi lines used as follows: dIDH2-KD1 (mild), dIDH2-KD2 (intermediate), and dIDH2-KD3 (severe). They also show that dIDH2 is a major regulator of OXPHOS assembly. Therefore, the three transgenic RNAi constructs provide an opportunity to mechanistically delineate the nature of the OXPHOS damage triggered by the loss of dIDH2 in adult thoracic muscles of *Drosophila*.

### RNAi-mediated disruption of dIDH2 activates ferroptotic signals

**Fig. 2.**
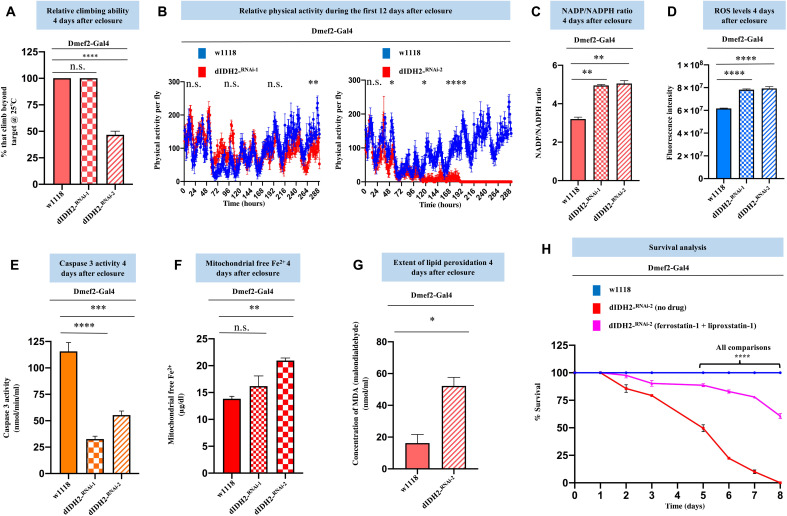
RNAi-mediated disruption of dIDH2 activates ferroptotic signals. (**A**) Relative climbing ability of flies with the genotypes shown. *n* = 3 biological replicates with 25 flies per replicate. (**B**) Relative spontaneous physical activities of dIDH2-KD1, dIDH2-KD2, and wild-type flies during the first 12 days after eclosure. *n* = 8 flies. (**C** to **G**) NADP:NADPH ratio (C), relative ROS levels (D), relative caspase 3 activity levels (E), relative mitochondrial free ferrous iron levels (F), and extent of lipid peroxidation (G) of dIDH2-KD1, dIDH2-KD2, and wild-type flies 4 days after eclosure. (**H**) Survival curves of dIDH2-KD2, wild-type flies, and dIDH2-KD2 flies raised on a diet supplemented with 1 mg/ml each of two ferroptosis inhibitors (ferrostatin-1 and liproxstatin-1). *n* = 3 biological replicates with 25 flies per replicate. In all instances except (B), (G), and (H), *P* values are based on one-way analysis of variance (ANOVA) followed by Dunnett’s multiple comparisons test. In (B), *P* values are based on two-way ANOVA followed by Sidak multiple comparisons test. In (G), *P* values are based on Student’s *t* test for unpaired two-tailed samples. One-way ANOVA followed by Tukey’s multiple comparisons test was used in (H). The fold change shown refers to the mean ± SEM, and n.s. denotes *P* > 0.05; *P < 0.05, **P < 0.01, ***P < 0.001, and ****P < 0.0001. The number of replicates (*n*) = 3 biological replicates with 10 flies per replicate in (C) to (E). There were 40 and 30 flies per replicate in (F) and (G), respectively.

To explore the functional consequences of disrupting dIDH2 expression, we examined the effect of knocking down dIDH2 in flight muscles on the locomotory activity of the flies. Overt climbing defects were observed in the dIDH2-KD2 flies (Fig. 2A). To further validate the locomotory defects observed in Fig. 2A, we monitored the spontaneous locomotory activity of dIDH2-KD1 and dIDH2-KD2 flies relative to wild-type flies (*Dmef2-Gal4/w^1118^)* within the first 2 weeks after the flies eclosed as adults. When analyzed at 25°C, the spontaneous locomotory ability of dIDH2-KD2 flies was markedly impaired starting around 48 hours after eclosure but occurring more consistently after about 120 hours after eclosure. This phenotype was less pronounced for the dIDH2-KD1 flies that did not exhibit a readily perceptible climbing defect until around 280 hours after eclosure (Fig. 2, A and B). The NADP^+^:NADPH ratio was increased in flight muscles from dIDH2-KD1 and dIDH2-KD2 flies, in agreement with the established function of IDH2 in reducing NADP^+^ to NADPH (Fig. 2C). An Amplex Red assay revealed that there was an increase in the amount of hydrogen peroxide—an indication of oxidative stress—in both dIDH2-KD1 and dIDH2-KD2 samples relative to wild-type controls (Fig. 2D). Despite the increased oxidative stress, the absence of caspase activation suggested that apoptosis was not induced (Fig. 2E). In addition, we found that the amount of labile ferrous iron was increased in the mitochondrion of dIDH2-KD2 flies relative to wild-type controls (Fig. 2F). As there was an increase in oxidative stress and ferrous iron levels, but caspase activity was not elevated in flight muscles from dIDH2-KD2 flies, we wondered whether ferroptosis was induced under these conditions. Thus, we examined whether the extent of lipid peroxidation in dIDH2-KD2 thoraces was increased relative to wild-type flies. Lipid peroxidation can culminate in the formation of thiobarbituric acid reactive substances (TBARS), which can be detected by a TBARS assay (see Materials and Methods). The TBARS assay revealed that the extent of lipid peroxidation was increased in dIDH2-KD2 samples (Fig. 2G). Raising the dIDH2-KD2 flies on a diet supplemented with two inhibitors of ferroptosis—ferrostatin-1 and liproxstatin-1—potently rescued the early lethality of dIDH2-KD2 flies (Fig. 2H). Thus, an integration of the bioenergetic and functional data shown in Figs. 1 and 2 indicates that disruption of dIDH2 impairs OXPHOS assembly and causes an up-regulation of several proferroptotic signals.

### Ferroptosis is induced in the flight muscles of dIDH2-KD2 flies

**Fig. 3.**
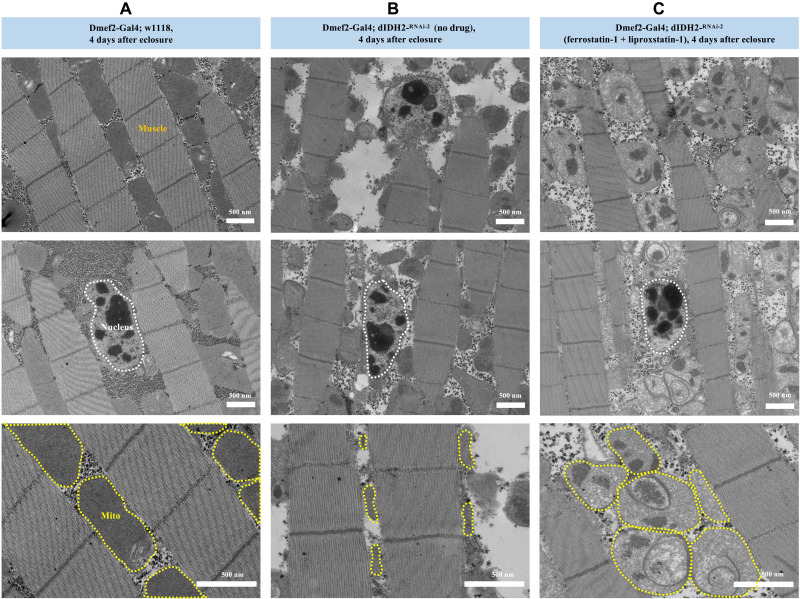
Ferroptosis is induced in the flight muscles of dIDH2-KD2 flies. Transmission electron micrographs of flight muscle sections from wild-type (**A**), dIDH2-KD2 (**B**), and dIDH2-KD2 flies raised on 1 mg/ml each of ferrostatin-1 and liproxstatin-1 (**C**). Note that, while the nucleus is intact in all three genotypes, shrunken mitochondria with increased membrane density are observed in the dIDH2-KD2 samples, which is suppressed by ferrostatin-1 and liproxstatin-1. However, because ferrostatin-1 and liproxstatin-1 rescue ferroptotic signals but do not restore NADPH biosynthesis in the dIDH2-KD2 flies, the mitochondrial integrity of dIDH2-KD2 flies raised on ferrostatin-1 and liproxstatin-1 is not restored to a wild-type state. See fig. S4 for data on rescue experiments with NADPH, ferrostatin-1, and liproxstatin-1.

Because multiple ferroptotic signals are induced in dIDH2-KD2 flight muscles, we wondered whether this results in ferroptotic cell death. In view of that, we performed transmission electron micrographs to assess the integrity of flight muscles from wild-type and dIDH2-KD2 flies (Fig. 3, A and B). We observed the hallmarks of ferroptosis of preserved nuclear integrity and shrunken mitochondria with increased membrane density in dIDH2-KD2 flight muscles (Fig. 3, A and B) (*25*–*27*). Importantly, raising the dIDH2-KD2 flies on ferrostatin-1 and liproxstatin-1 potently rescued these morphological features (Fig. 3C). There were no overt changes in myofiber integrity in dIDH2-KD2 flies, indicating that the deleterious phenotypes are largely due to mitochondrial dysfunction. In addition, the preservation of nuclei in dIDH2-KD2 flight muscles is consistent with our caspase activity results in Fig. 2E, indicating that apoptosis is not induced in dIDH2-KD2 flight muscles (Fig. 3, A to C). Because raising the flies on up to 10 mM NADPH did not rescue the OXPHOS assembly defect (fig. S4), we examined the effect of suppressing ferroptosis on the OXPHOS assembly defect. Raising the flies on ferrostatin-1 and liproxstatin-1 did not rescue the impaired OXPHOS assembly phenotype in the flight muscles of dIDH2-KD2 flies (fig. S4). A plausible explanation for this results is that ferroptosis may occur downstream of the respiratory complex defects.

### The synthesis of matrix-localized CI assembly intermediates is impaired when dIDH2 is disrupted

**Fig. 4.**
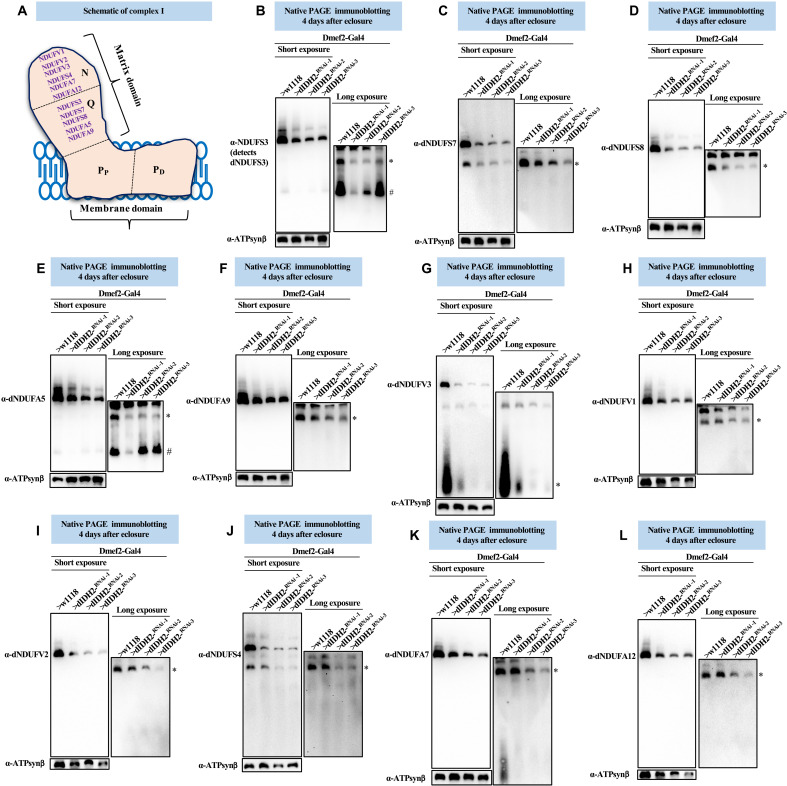
The synthesis of matrix-localized CI AIs is impaired when dIDH2 is disrupted. (**A**) Illustration of mitochondrial CI depicting the approximate positions of the N-, Q-, P_P_-, and P_D_-modules. A commercially available antibody that detects dNDUFS3 and antibodies we raised against dNDUFA5, dNDUFA9, dNDUFS7, and dNDUFS8 were used to examine the biogenesis of the Q-module. Biogenesis of the N-module was tracked by immunoblotting with antibodies we raised against dNDUFS4, dNDUFA7, dNDUFA12, dNDUFV1, dNDUFV2, and dNDUFV3. (**B** to **L**) Mitochondrial preparations from flight muscles dissected from flies with the genotypes shown were analyzed by BN-PAGE, followed by immunoblotting with the antibodies indicated. The blots were imaged following a short exposure to detect the holoenzyme and supercomplexes, after which the region corresponding to the holoenzyme and supercomplexes was cut off, and the rest of the blot was reimaged after a longer exposure to detect the AIs. The antibodies used were anti-NDUFS3 that detects dNDUFS3 (B), anti-dNDUFS7 (C), anti-dNDUFS8 (D), dNDUFA5 (E), anti-dNDUFA9 (F), anti-dNDUFV3 (G), anti-dNDUFV1 (H), anti-dNDUFV2 (I), anti-dNDUFS4 (J), anti-dNDUFA7 (K), and anti-dNDUFA12 (L). Anti–ATP synthase, subunit B (ATP5F1B), which detects the *Drosophila* ortholog, dATP-Synβ, was used as a loading control. # refers to the initiating AI of the Q-module, and other AIs are denoted as *. AIs denoted with # and * were quantified. See figs. S5 to S7 for replicates and quantification of immunoblots.

Mitochondrial CI has a characteristic L-shaped structure. It consists of a matrix domain extending into the mitochondrial matrix, oriented almost perpendicularly to a membrane domain localized to the mitochondrial inner membrane (Fig. 4A) (*28*, *29*). To begin to uncover the mechanism by which dIDH2 regulates OXPHOS assembly, we examined the effect of disrupting dIDH2 on the biogenesis of the matrix domain of CI (Fig. 4A). During CI assembly, specific assembly intermediates (AIs) consisting of a few CI subunits form largely independently of each other and merge in a stereotypic fashion en route to forming the mature complex (*3*, *16*, *30*). The N-, Q-, and P-modules are synthesized from specific AIs.

Synthesis of the Q-module begins with the formation of an AI consisting of dNDUFS2 and dNDUFS3, which ultimately combines with dNDUFS7, dNDUFS8, dNDUFA5, and dNDUFA9. As a consequence, we tracked the biogenesis of the Q-module via immunoblotting of blue native gels with antibodies that detect dNDUFS3, dNDUFS7, dNDUFS8, dNDUFA5, and dNDUFA9. There was a reduction in the amount of dNDUFS3 in an initiating AI of the Q-module in mitochondria isolated from flight muscles of dIDH2-KD1 thoracic samples (Fig. 4B). Moreover, the amount of dNDUFS3 that had incorporated into a more advanced NDUFS3-containing AI was diminished in dIDH2-KD1, dIDH2-KD2, and dIDH2-KD3 samples. Similarly, the amount of dNDUFS7, dNDUFS8, dNDUFA5, and dNDUFA9 that had incorporated into the Q-module was reduced as a result of dIDH2 disruption (Fig. 4, C to F).

The N-module consists of the following subunits: dNDUFV1, dNDUFV2, dNDUFV3, dNDUFA2, dNDUFA6, dNDUFA7, dNDUFA12, dNDUFS1, dNDUFS4, and dNDUFS6. Hence, we tracked the incorporation of dNDUFV1, dNDUFV2, dNDUFV3, dNDUFS4, dNDUFA7, and dNDUFA12 into CI AIs to monitor the integrity of the N-module. We observed that the stabilization or incorporation of dNDUFV3 into an initiating AI of the N-module was impaired in mitochondria isolated from flight muscles of dIDH2-KD1, dIDH2-KD2, and dIDH2-KD3 flies (Fig. 4G). This was coupled with a decrease in the amount of dNDUFV1, dNDUFV2, dNDUFS4, dNDUFA7, and dNDUFA12 that had incorporated into subcomplexes of the N-module isolated from mitochondria obtained from all three RNAi backgrounds, but most notably from the dIDH2-KD2 and dIDH2-KD3 flies (Fig. 4, H to L). The AI profile observed in the matrix domain as a result of disrupting dIDH2 expression was reproducible (figs. S5 to S7). Together, we conclude that disruption of IDH2 impairs the biogenesis or stability of the Q- and N-modules.

### Biogenesis of some membrane domain CI AIs is stalled due to disruption of dIDH2

**Fig. 5.**
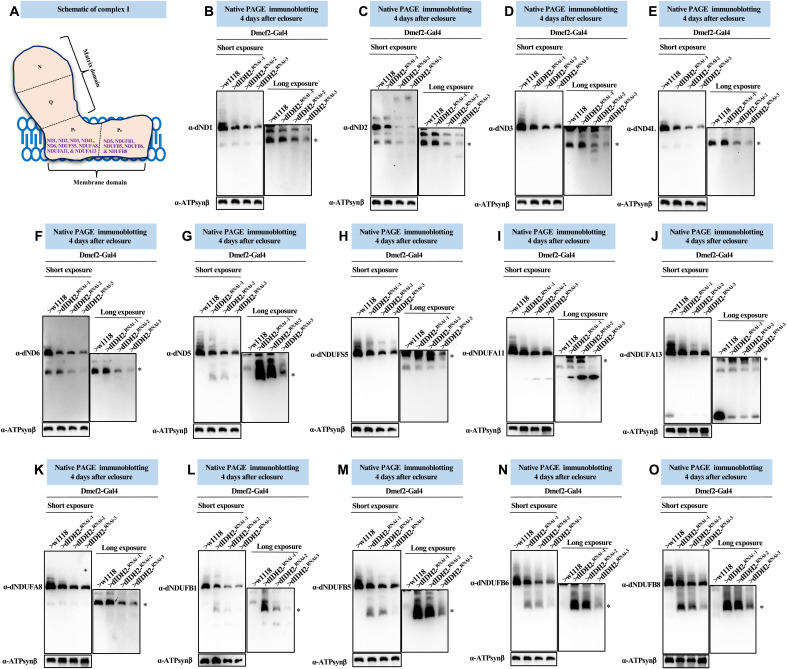
Biogenesis of some membrane domain CI AIs are stalled due to disruption of dIDH2. (**A**) Diagram of mitochondrial CI showing the antibodies used to monitor the synthesis of the P_P_- and P_D_-submodules. (**B** to **O**) Mitochondrial preparations from flight muscles isolated from Dmef2-Gal4/w^1118^ (wild-type), dIDH2-KD1, dIDH2-KD2, and dIDH2-KD3 flies 4 days after eclosure were analyzed by BN-PAGE, followed by immunoblotting with the antibodies listed. The antibodies used were anti-dND1 (B), anti-dND2 (C), anti-dND3 (D), anti-dND4L (E), anti-dND6 (F), anti-dND5 (G), anti-dNDUFS5 (H), anti-dNDUFA11 (I), anti-dNDUFA13 (J), anti-dNDUFA8 (K), anti-dNDUFB1 (L), anti-dNDUFB5 (M), anti-dNDUFB6 (N), and anti-dNDUFB8 (O). Anti–ATP synthase, subunit B (ATP5F1B) was used as a loading control. See figs. S8 to S10 for replicates and quantification of immunoblots.

The membrane domain is composed of the proton-pumping P-module, which can be further subdivided into a proximal P_P_-module and a distal P_D_-module. Five of seven mitochondrial DNA (mtDNA)–encoded subunits (i.e., dND1, dND2, dND3, dND4L, and dND6) are part of the P_P_-module, while dND4 and dND5 are localized to the P_D_-module (Fig. 5A) [reviewed in (*3*, *31*)].

We explored whether disruption of dIDH2 impairs the incorporation of the mtDNA-encoded CI subunits into the P-module (Fig. 5A). The incorporation of dND1 into the P_P_-module was found to be progressively more impaired in dIDH2-KD1, dIDH2-KD2, and dIDH2-KD3 samples, respectively (Fig. 5B). Similarly, incorporation of dND2 was not appreciably perturbed in dIDH2-KD1 flight muscles but showed a modest trend toward reductions in dIDH2-KD2 and dIDH2-KD3 samples (Fig. 5C). We previously showed that dND3, dND4L, and dND6 are unstable and rapidly degraded when CI biogenesis is impaired (*17*). A dND3-containing AI was not appreciably stalled in dIDH2-KD1 samples, but we observed what appeared to be a smear of the dND3-containing AI in dIDH2-KD2 samples, suggesting that the AI had stalled and was probably undergoing degradation (Fig. 5D). AIs containing dND4L and dND6 were also not appreciably perturbed in dIDH2-KD1 flight muscles but were progressively more reduced in dIDH2-KD2 and dIDH2-KD3 samples, respectively (Fig. 5, E and F). Finally, a dND5-containing AI was robustly stalled in dIDH2-KD1 and dIDH2-KD2 samples but only modestly in dIDH2-KD3 samples, although there appeared to be remnants of a dND5-containing AI that had degraded in the dIDH2-KD3 samples (Fig. 5G).

We also examined the effect of knocking down dIDH2 expression on the incorporation of nuclear-encoded CI subunits into the P-module (Fig. 5A). Incorporation of dNDUFS5 and dNDUFA11 appeared to accumulate in a terminal AI that migrates just below the CI holoenzyme in dIDH2-KD1 and dIDH2-KD2 flight muscles, but the amount in dIDH2-KD3 samples was reduced (Fig. 5, H and I). Similarly, there was some evidence of a stalling and accumulation of a dNDUFA13-containing AI in dIDH2-KD1 and dIDH2-KD2 flight muscles, but not in the dIDH2-KD3 sample (Fig. 5J). Furthermore, incorporation of dNDUFA8 was also disrupted as a result of dIDH2 knockdown (Fig. 5K). In line with observations described in Fig. 5G, immunoblotting against multiple P_D_-module subunits (i.e., dNDUFB1, dNDUFB5, dNDUFB6, and dNDUFB8) showed that the P_D_-module was severely stalled in dIDH2-KD1 and dIDH2-KD2 samples (Fig. 5, L to O). In essentially all instances where a P-module AI accumulated in dIDH2-KD1 or dIDH2-KD2 flight muscles but not in dIDH2-KD3 flight muscles, there appeared to be vestiges of the accumulated AI in dIDH2-KD3. This finding may be indicative of an initial accumulation of the AI in dIDH2-KD3 that eventually succumbed to proteolytic degradation (Fig. 5, G and L to O). We note that the AI profile observed in the membrane domain as a result of disrupting dIDH2 expression was largely reproducible (figs. S8 to S10). Collectively, the results described herein point to the following: There was a stalling and accumulation of many P_D_-module AIs that was readily apparent in the dIDH2-KD1 samples but was sometimes observed in the dIDH2-KD2 and dIDH2-KD3 samples as well. In some instances, subunits in the accumulated AI appeared to be undergoing degradation. This was most noticeable in dIDH2-KD3 samples. When combined with results from Fig. 4, we infer that biogenesis of the P-module was probably not as substantially inhibited as the Q- and N-modules when dIDH2 is disrupted. However, because the progression of the P-module in the CI biogenesis pathway is dependent on an intact Q-module, the reduction in synthesis or stability of the Q-module when dIDH2 is genetically impaired appears to cause a stalling and accumulation of multiple AIs in the P-module, which ultimately succumb to degradation.

### A compensatory response is induced as a result of genetic disruption of dIDH2

**Fig. 6.**
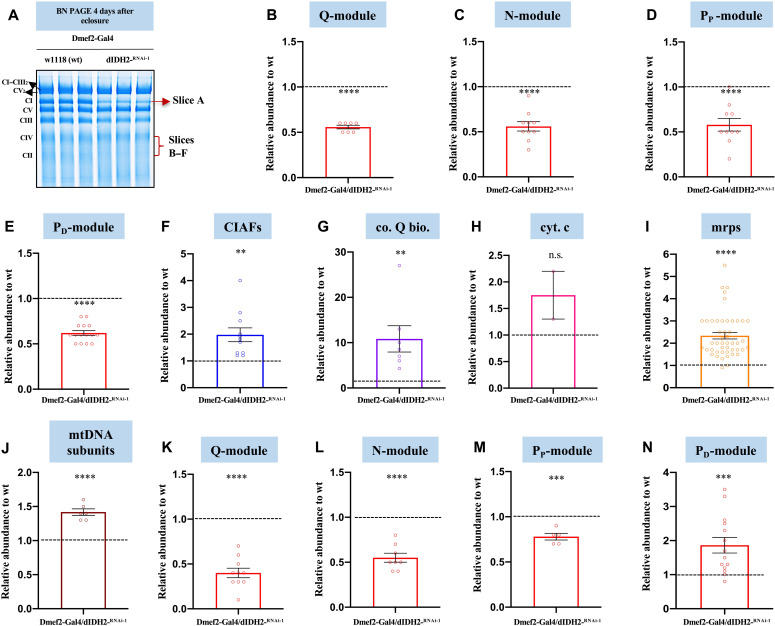
A compensatory response is induced as a result of genetic disruption of dIDH2. (**A**) BN-PAGE gel showing the gel slices that were excised and analyzed by mass spectrometry. (**B** to **E**) Label-free quantitative proteomics was used to assess the relative amount of CI subunits in the holoenzyme (gel slice A) that are part of the Q-module (B), N-module (C), P_P_-module (D), and P_D_-module (E) in mitochondrial preparations from Dmef2-Gal4/w^1118^ (wild-type) and dIDH2-KD1 fly thoraces. (**F** to **J**) Label-free quantitative proteomics was used to quantify the relative amount of CIAFs (F), genes that regulate coenzyme Q biosynthesis (co. Q bio.) (G), cytochrome c (cyt. c) (H), mitochondrial ribosomal proteins (mrps) (I), and mtDNA-encoded OXPHOS subunits (J), which comigrate with the holoenzyme in mitochondrial preparations from Dmef2-Gal4/w^1118^ (wild-type) and dIDH2-KD1 fly thoraces. (**K** to **N**) Label-free quantitative proteomics was used to assess the relative amount of CI subunits in gel slice B (overlaps with CIV) that are part of the Q-module (K), N-module (L), P_P_-module (M), and P_D_-module (N). In all six gel slices, *n* = 2 biological replicates with mitochondria from 20 fly thoraces per replicate; *P* values are based on Student’s *t* test for unpaired two-tailed samples. The fold change shown refers to the mean ± SEM, and n.s. denotes *P* > 0.05; **P < 0.01, ***P < 0.001, and ****P < 0.0001.

We used a proteomics approach to gain further insight into the mechanism by which disruption of dIDH2 affects the OXPHOS. Specifically, we isolated mitochondria from dIDH2-KD1 and wild-type flight muscles, solubilized their mitochondrial membranes in digitonin, and resolved their OXPHOS complexes by BN-PAGE. Subsequently, we excised a portion of the blue native gel that encompasses the CI holoenzyme (hereinafter referred to as profile A), extracted the constituent proteins, and analyzed them by mass spectrometry (MS) (Fig. 6A). We used label-free spectral counting to assess the relative abundance of individual subunits in the various CI modules of the holoenzyme from dIDH2-KD1 flight muscles relative to the wild-type sample (*32*–*34*).

As anticipated, CI subunits in the Q-, N-, P_P_-, and P_D_-modules were all down-regulated in the gel slice corresponding to the holoenzyme (Fig. 6, B to E, and table S1). Several CI assembly factors (CIAFs) remained in association with the residual CI in the dIDH2-KD1 samples (Fig. 6F and table S1). As many CIAFs function as chaperones to stabilize relatively unstable CI AIs, their association with the CI that remains in dIDH2-KD1 flight muscles appears to be an adaptive response to preserve a substoichiometric amount of CI. Notably, there was a robust and statistically significant up-regulation of multiple proteins involved in coenzyme Q biosynthesis, which comigrated with the residual CI in dIDH2-KD1 flight muscles (Fig. 6G). In addition, the two paralogs of cytochrome c in *Drosophila* showed a trend toward up-regulation, although the changes were not statistically significant (Fig. 6H and table S1). There was also a modest but consistent increase in the amount of several mitochondrial ribosomal proteins comigrating with CI in dIDH2-KD1 samples (Fig. 6I and table S1). As translation of mtDNA-encoded CI subunits and subsequent integration into the CI biosynthetic pathway occur almost concurrently, these observations are consistent with the induction of a robust compensatory mitochondrial translation response aimed at promoting OXPHOS biogenesis under suboptimal conditions. All other mtDNA-encoded OXPHOS subunits [mt: cyt-b in CIII; mt: coI, mt: coII, and mt: coIII in CIV; and mt: adenosine triphosphatase (ATPase) 6 and mt: ATPase 8 in CV] were up-regulated (Fig. 6J and table S1). Together, these results allude to the induction of a robust adaptive response in dIDH2-KD1 flight muscles aimed at ensuring the continuous passage of electrons through the electron transport chain, in spite of a suboptimal CI.

We previously showed that most of the initiating AIs migrate at a region between CII and CIV in blue native gels (*16*). Therefore, we divided the region of the blue native gel between CII and CIV into five evenly sized slices, labeled slices B to F, from higher to lower molecular weight and analyzed their proteomic constituents by MS (Fig. 6A). Multiple Q-, N-, and P_P_-module subunits, as well as the CIAFs—dNDUFAF3 and dNDUFAF4—were down-regulated in the dIDH2-KD1 sample in profile B (Fig. 6, K to M, and table S2). However, most P_D_-module subunits were up-regulated in profile B (Fig. 6N and table S2). Altogether, the wave of CI biogenesis observed in profile B indicates that initiating Q-, N-, and P_P_-module AIs were unstable in the dIDH2-KD1 sample, resulting in a stalling and accumulation of the P_D_-module, as the P_D_-module can only progress further in the CI biosynthesis pathway if there are sufficient “binding partners” of the P_P_-module. Interestingly, this conclusion is in harmony with our immunoblotting results described in Figs. 4 and 5. Our proteomic studies also suggested that an induction of genes that regulate the mitochondrial unfolded protein response (UPR^mt^) and possibly other regulators of mitochondrial protein homeostasis may be a prominent component of the mitochondrial stress signaling network activated as a result of disrupting dIDH2 (tables S1 to S6). In aggregate, these data strongly suggest that a compensatory adaptive response aimed at repairing or degrading misfolded proteins in the mitochondrion is induced when dIDH2 is disrupted.

### RNA sequencing analyses identify genes induced in dIDH2-KD2 flight muscles as an adaptive response

**Fig. 7.**
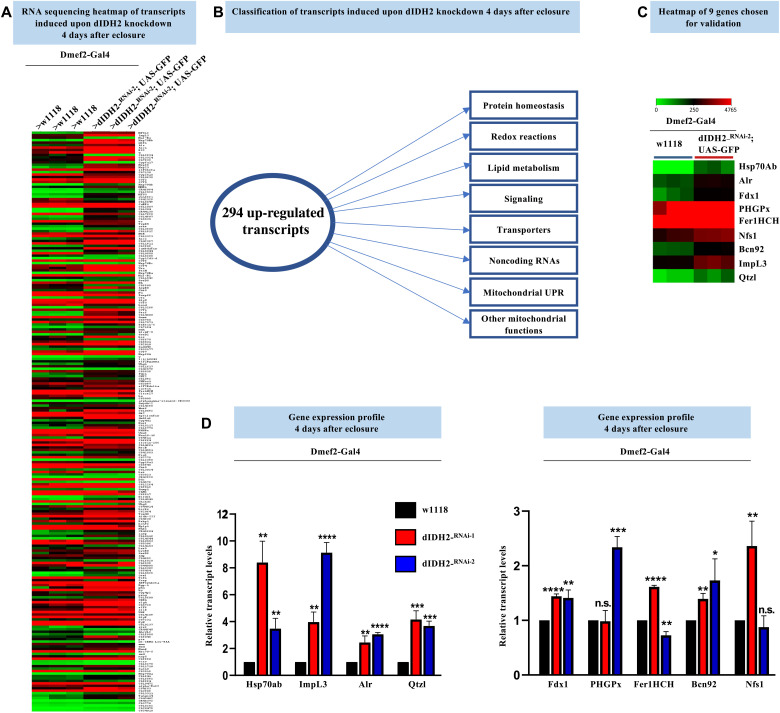
RNA-seq analyses identify genes induced in dIDH2-KD2 thoraces as an adaptive response. (**A**) Heatmap depicting the transcripts that are up-regulated or down-regulated by 1.5-fold (log_2_ ratio of 0.6) or more in Dmef2-Gal4/UAS-dIDH2^-RNAi-2^;UAS-GFP thoraces relative to Dmef2-Gal4/w^1118^ (wild-type) thoraces. (**B**) Schematic summarizing the major gene classes or cellular processes regulated by the 294 genes induced in Dmef2-Gal4/UAS-dIDH2^-RNAi-2^;UAS-GFP thoraces. (**C**) Smaller heatmap of a set of up-regulated genes chosen for confirmation by quantitative RT-PCR in (D). (**D**) Relative transcript levels of the genes shown in (C), as assessed by quantitative RT-PCR. To assess the significance of the gene expression results, *P* values were computed on the basis of one-way ANOVA followed by Dunnett’s multiple comparisons test. The fold change shown refers to the mean ± SEM, and n.s. denotes *P* > 0.05; *P < 0.05, **P < 0.01, ***P < 0.001, and ****P< 0.0001. The number of replicates (*n*) = 3 biological replicates with 10 flies per replicate.

To obtain a more comprehensive view of the signaling events and pathways induced in dIDH2-KD2 flight muscles, we used RNA sequencing (RNA-seq). We surveyed the whole transcriptome of flight muscles from dIDH2-KD2 flies overexpressing green fluorescent protein (GFP) (i.e., UAS-GFP). The UAS-GFP transgene was included as a negative control to allow comparisons with dIDH2-KD2 flight muscles overexpressing or knocking down other transgenes in subsequent studies (not in this article). We dissected the dIDH2-KD2/UAS-GFP flight muscles 4 days after eclosure and compared their gene expression profile to those of age-matched wild-type controls (Fig. 7A). A total of 294 transcripts were found to be induced by 1.5-fold or more in the dIDH2-KD2/UAS-GFP samples relative to the wild-type control (adjusted *P* value < 0.05) (Fig. 7B and table S7). Many genes implicated in regulating the UPR^mt^ or general mitochondrial protein homeostasis were up-regulated in response to down-regulation of dIDH2. These include *hsp60A* (CG12101), one of the two *Drosophila* paralogs of *hsp10* (CG11267), *PMPCA* (CG8728), *AFG3L2* (CG6512), and the mitochondrial hsp70 isoform *hsc70-5* (CG8542). Multiple genes implicated in the de novo synthesis of iron-sulfur (Fe-S) clusters such as *Fdx1* (CG4205), *Nfs1* (CG12264), *LYRM4/bcn92* (CG3717), and *Qtzl* (CG31864), a paralog of Nfs1, were also induced in the dIDH2-KD2/UAS-GFP samples (Fig. 7C). The up-regulation of genes that regulate Fe-S cluster biogenesis is consistent with an adaptive compensatory response, as Nfs1 has been shown to protect cells from undergoing ferroptosis (*35*). Alr, the *Drosophila* ortholog of Erv1 in *Saccharomyces cerevisiae* and a component of the mitochondrial intermembrane space assembly (MIA) pathway, was also induced when dIDH2 was disrupted, perhaps as a compensatory response to a suboptimally functioning MIA system. Transcripts encoding ferritin and the two *Drosophila* paralogs encoding *GPX4* (PHGPx and CG15116) were also induced—possibly as part of an adaptive response to the ferroptotic signals induced when dIDH2 is disrupted (table S7). Lactate dehydrogenase (Impl3, CG10160) was also up-regulated (table S7). We confirmed the up-regulation of most of these genes using quantitative reverse transcription polymerase chain reaction (RT-PCR) (Fig. 7D and Table 1). Overall, the major classes of induced genes included regulators of general protein homeostasis, mitochondrial function, lipid metabolism, redox reactions, signaling pathways, and DNA repair/synthesis as well as transporters and noncoding RNAs (Fig. 7B). Taken together, the RNA-seq results reinforce our conclusion that a compensatory adaptive response aimed at repairing the damage caused by the ferroptotic signals is induced when dIDH2 is disrupted.

**Table 1.** A sample of genes induced as a result of disrupting dIDH2 function in thoraces.

**Gene**	**Fold ** **change ** **(linear)**	**Adjusted *P* value**	**Human/yeast ** **ortholog**	**Gene group**	**Cellular component**	**Biological process**
Hsp70Ab (CG18743)	402.57	4.21 × 10^−9^	HSPA1A/SSA3	Heat shock protein 70 chaperone	Cytosol, nucleus, membrane, cell periphery	Multiple functions related to protein homeostasis, response to stress
Alr (CG12534)	3.82	1.05 × 10^−16^	GFER/ERV1	Sulfur oxidoreductase	Mitochondrion	Mitochondrial protein homeostasis, flavin adenine dinucleotide binding activity
Fdx1 (CG4205)	2.54	0.001743	FDX2/YAH1	Oxidoreductase	Mitochondrion	Ferredoxin 1, required for iron-sulfur cluster biogenesis and ecdysteroidogenesis
PHGPx (CG12013)	2.39	0.004599	GPX4/GPX2	Glutathione peroxidase	Endomembrane system	Antioxidant enzyme, prevents lipid peroxidation, response to stress
Fer1HCH (CG2216)	2.30	0.002471	FTH1/none	Ferritin	Cytosol, endomembrane system	Ferritin 1 heavy chain homolog, major iron storage complex, ferroxidase activity
Bcn92 (CG3717)	1.91	0.013529	LYRM4/ISD11	Complex I LYR family	Cytosol, mitochondrion	Part of cysteine desulfurase complex, regulates iron-sulfur cluster assembly
Nfs1 (CG12264)	2.12	0.008541	NFS1/NFS1	Sulfurtransferase	Cytosol, mitochondrion, nucleus	Cysteine desulfurase, regulates Fe-S cluster biogenesis
Qtzl (CG31864)	3.03	0.025038	NFS1/NFS1	Sulfurtransferase	Cytosol, mitochondrion, nucleus	Paralog of CG12264, cysteine desulfurase, regulates Fe-S cluster biogenesis
ImpL3 (CG10160)	4.09	1.88 × 10^−20^	LDHA/MDH1	LDH/MDH superfamily	Largely cytosolic	Lactate dehydrogenase, catalyzes the interconversion between lactate and pyruvate

### The UPR^mt^ is induced as part of an adaptive ISR in dIDH2-KD1 and dIDH2-KD2 flight muscles

The rotenone-insensitive NADH-ubiquinone oxidoreductase (NDI1) is a nuclear-encoded polypeptide in yeast that transfers electrons to ubiquinone. In this respect, it compensates for the lack of a multisubunit CI in yeast, although it lacks the proton-pumping ability of CI. Overexpression of the NDI1 enzyme in *Drosophila* leads to an increase in NADH-ubiquinone oxidoreductase activity and decreases ROS levels (*36*–*39*). Accordingly, we examined the effect of overexpressing NDI1 in dIDH2-KD2 flight muscles.

**Fig. 8.**
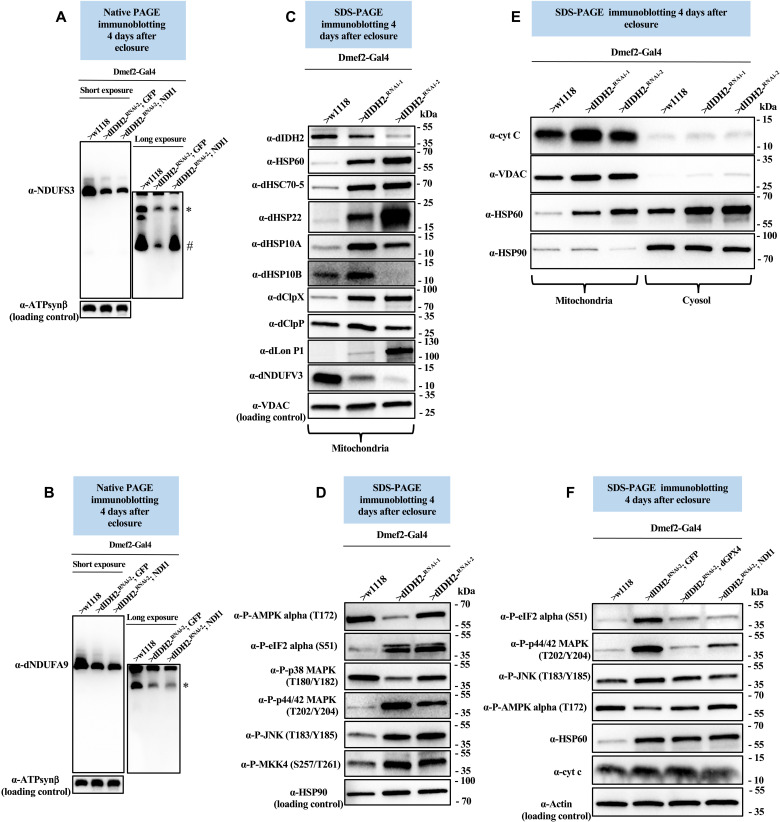
The UPR^mt^ is induced as part of an adaptive integrated stress response in dIDH2-KD1 and dIDH2-KD2 flight muscles. (**A** and **B**) Flight muscle mitochondrial preparations from flies with the genotypes shown were analyzed by BN-PAGE, followed by immunoblotting with anti-NDUFS3 (A) and anti-dNDUFA9 (B). # refers to the initiating AI of the Q-module, and other AIs are denoted as *. AIs denoted with # and * were quantified. (**C**) Flight muscle mitochondrial lysates from flies with the genotypes shown were analyzed by SDS-PAGE and immunoblotting with the antibodies indicated, most of which detect mitochondrial chaperones and proteases. (**D**) Flight muscle total tissue lysates from flies with the genotypes shown were analyzed by SDS-PAGE and immunoblotting with the antibodies indicated, most of which detect stress-activated kinases. (**E**) Mitochondrial preparations (left three lanes) and cytosolic fractions (right three lanes) from flight muscles of flies with the genotypes listed were analyzed by SDS-PAGE and immunoblotting with the antibodies indicated. Anti-VDAC and anti-Hsp90 were used as loading controls for the mitochondrial and cytosolic fractions, respectively. (**F**) Total tissue lysates from flight/thoracic muscles isolated from flies with the genotypes listed were analyzed by SDS-PAGE and immunoblotting with the antibodies indicated. See figs. S11 to S15 for replicates and quantification of immunoblots. Loading controls used are as indicated.

We compared CI AI profiles in flight muscle mitochondrial proteins from dIDH2-KD2 flies that overexpress GFP (dIDH2-KD2/UAS-GFP, negative control) and dIDH2-KD2 flies that overexpress NDI1 (dIDH2-KD2 RNAi/UAS-NDI1). Similar to our findings in Fig. 4B, biogenesis or stability of the Q-module, assessed by immunoblotting of dNDUFS3, was impaired in dIDH2-KD2/UAS-GFP flight muscles. In contrast, overexpression of NDI1 in dIDH2-KD2 flight muscles led to a notable restoration of the dNDUFS3-containing initiating AI in flight muscles (Fig. 8A). However, despite this rescue in the dNDUFS3-containing AI, NDI1 overexpression was not sufficient to ameliorate the diminished incorporation of dNDUFA9 into the matrix domain (Fig. 8B). As a result, forced expression of NDI1 in dIDH2-KD2 flight muscles restores the initial synthesis or stability of the Q-module but not the remainder of the matrix domain.

To explore the effect of overexpressing NDI1 on the mitochondrial stress signaling network activated in dIDH2-KD1 flight muscles, we first characterized the mitochondrial stress signaling cascade induced as a result of disrupting IDH2. Mitochondrial dysfunction activates a conserved stress signaling cascade termed the UPR^mt^ (*40*, *41*). The mitochondrial chaperones Hsp60, mtHsp70, and Hsp10 as well as the Clp protease have all been associated with the UPR^mt^ (*40*, *41*). The Clp protease consists of hexamers of a AAA+ ATPase (ClpX) and a tetradecameric peptidase (ClpP). Lon protease (LONP1) is known to degrade oxidatively damaged proteins in the mitochondrial matrix (*42*). The mitochondrial chaperone Hsp22 is also potently induced in *Drosophila* tissues in response to mitochondrial distress (*43*). Our RNA-seq dataset indicated that induction of genes that regulate the UPR^mt^, and possibly other regulators of mitochondrial protein homeostasis, may be a prominent component of the mitochondrial stress signaling network activated as a result of disrupting dIDH2 (table S7). Therefore, we generated antibodies to the *Drosophila* ortholog of mtHsp70 (Hsc70-5), Hsp10 (both paralogs), Hsp22, ClpP, ClpX, and Lon protease and used immunoblotting of proteins extracted from flight muscle mitochondria to assess the relative expression of proteins implicated in mitochondrial protein homeostasis in dIDH2-KD1, dIDH2-KD2, and wild-type samples. Although dNDUFV3 expression was reduced in the mitochondrion—a reflection of the disintegration and degradation of CI—there was a potent up-regulation of proteins that regulate the UPR^mt^ (Fig. 8C and figs. S11 to S13). These results indicate that disruption of dIDH2 enhances the UPR^mt^ possibly to curb the damaging effects of oxidative stress on proteins.

When exposed to diverse stress stimuli, eukaryotic cells activate a common adaptive response, referred to as the ISR, to restore cellular homeostasis. A major event in this signaling cascade is the global down-regulation of protein synthesis and the induction of a select set of cytoprotective genes that act in unison to promote cellular recovery. Phosphorylation of the α subunit of eukaryotic initiation factor 2 (eIF2α) is an established mechanism for limiting protein synthesis under various stress conditions (*44*). Thus, we examined the extent of activation of various stress-responsive kinases and phospho-eIF2α (Ser^51^) (Fig. 8D). We were able to detect an increase in the amount of phospho-eIF2α, phospho-p44/42 mitogen-activated protein kinase (MAPK) (Erk1/2), phospho–stress-activated protein kinase/c-Jun N-terminal kinase (SAPK/JNK), phospho-SAPK/Erk kinase (SEK1), also known as mitogen-activated protein kinase kinase 4 (MKK4) in dIDH2-KD1 and dIDH2-KD2 flight muscles relative to wild-type samples, but both phospho-AMP-activated protein kinase (AMPK) and phospho-p38 appeared to be down-regulated in dIDH2-KD1 and dIDH2-KD2 flight muscles (Fig. 8D and figs. S11 and S12). Although the ISR is primarily an adaptive prosurvival response, the signaling cascade can become maladaptive with persistence of the stress exposure and can ultimately induce cell death. As a result, we examined whether cytochrome c was released from the mitochondrion as an indication of the initial steps of apoptosis. Cytochrome c levels were neither significantly increased in the mitochondrion nor released from the mitochondrion to cause apoptosis (Fig. 8E and figs. S11, S12, and S14). This is in line with results from Fig. 3 indicating that the myonuclei from dIDH2-KD2 flight muscles were preserved in contrast to what would be expected if apoptosis was induced.

Finally, we examined the effect of up-regulating NDI1 or the antioxidant enzyme (dGPX4) on the stress signaling pathways activated in dIDH2-KD2 flight muscles. Forced expression of NDI1 or dGPX4 dampened the extent of activation of the phospho-eIF2α, phospho-p44/42 MAPK (Erk1/2), and phospho-SAPK/JNK pathways (Fig. 8F and fig. S15). Nevertheless, the amount of Hsp60 or cytochrome c was not appreciably altered (Fig. 8F and fig. S15). We note, however, that there appeared to be oscillating phosphorylation-dephosphorylation cycles associated with the activation of most of these kinases, as has been discussed previously (figs. S14 and S15) (*45*–*47*). Nevertheless, we conclude from these results that the UPR^mt^ is induced as part of an ISR in dIDH2-KD1 and dIDH2-KD2 flight muscles.

### Disruption of dME3 impairs the biogenesis of CI AIs

Malic enzymes (MEs) catalyze the oxidative decarboxylation of malate to pyruvate in a process that results in the reduction of NADP^+^ to NADPH, or NAD^+^ to NADH. Similar to IDH, three ME isoforms exist. ME1 is localized to the cytosol, while ME2 and ME3 are located in the mitochondrion. ME1 and ME3 use NADP^+^ as a cofactor, while ME2 can use either NADP^+^ or NAD^+^. CG5889 (Men-b) is the *Drosophila* ortholog of ME3. As is evident from Fig. 1B, RNAi-mediated knockdown of dME3 causes a CI assembly defect. In view of the fact that both dME3 and dIDH2 reduce NADP^+^ to NADPH and the OXPHOS assembly defect observed when dME3 is knocked down is similar to that of dIDH2-KD1, we wondered whether RNAi-mediated knockdown of dME3 in flight muscles using the Dmef2-Gal4 transgene (i.e., dME3-KD) also impairs the biogenesis of CI AIs.

**Fig. 9.**
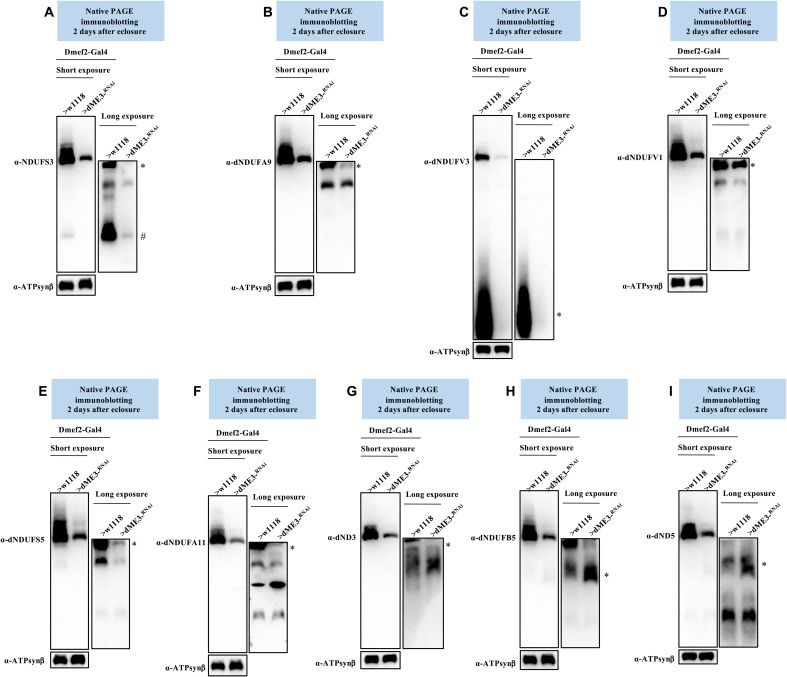
Disruption of dME3 impairs the biogenesis of CI AIs. Mitochondrial preparations from flight muscles isolated from Dmef2-Gal4/w^1118^ (wild-type) and Dmef2-Gal4/UAS-dME3^-RNAi^ flies 2 days after eclosure were analyzed by BN-PAGE, followed by immunoblotting with the antibodies listed. The antibodies used were anti-NDUFS3 (**A**), anti-dNDUFA9 (**B**), anti-dNDUFV3 (**C**) anti-dNDUFV1 (**D**), anti-dNDUFS5 (**E**), anti-dNDUFA11 (**F**), anti-dND3 (**G**), dNDUFB5 (**H**), and anti-dND5 (**I**). Anti–ATP synthase, subunit B was used as a loading control. AIs denoted with # and * were quantified. See figs. S16 to S18 for replicates and quantification of immunoblots.

Consequently, we examined the effect of disrupting dME3 on the biogenesis of the matrix domain of CI (Fig. 4A). There was a reduction in the amount of dNDUFS3 in an initiating AI of the Q-module in mitochondria isolated from flight muscles of dME3-KD flies (Fig. 9A). Similarly, the amount of dNDUFA9 that had incorporated into the Q-module was diminished in dME3-KD flight muscles (Fig. 9B). We also monitored the incorporation of dNDUFV1 and dNDUFV3 into CI AIs to ascertain the integrity of the N-module. We observed that the stabilization or incorporation of both dNDUFV3 and dNDUFV1 into some AIs of the N-module was diminished in mitochondria isolated from the flight muscles of dME3-KD flies (Fig. 9, C and D). This was coupled with a decrease in the amount of the P_P_-module subunits (dNDUFS5, dNDUFA11, and dND3) that had incorporated into subcomplexes from dME3-KD flight muscles (Fig. 9, E to G). Last, there was a slight stalling and accumulation of subunits in the P_D_-module, such as dNDUFB5 and dND5 (Fig. 9, H and I). We were able to reproduce the AI profile observed in the dME3-KD samples (figs. S16 to S18). We conclude that disruption of dME3 impairs the biogenesis or stability of the Q- and N-modules to trigger a disruption in CI assembly. Thus, mitochondrial NADPH-generating enzymes modulate the assembly of the matrix domain of CI.

## DISCUSSION

We found that when mitochondrial NADPH production is impaired as a result of dIDH2 disruption, ROS levels are elevated. This is associated with a deterioration of the OXPHOS complexes in a defined order. It commences with a disruption of CI assembly but progresses to the point where multiple OXPHOS complexes are impaired. NADPH is required for regenerating GSH in the glutathione cycle and for reactivating catalase (*48*). Consequently, given that the ratio of GSSG to GSH would likely increase when dIDH2 is knocked down, a plausible explanation for the increased ROS production is that insufficient amounts of GSH cause an increase in oxidative stress, which ultimately destabilizes the OXPHOS complexes. The fact that CI is the first complex to disintegrate when dIDH2 is impaired suggests that this is unlikely to be due to nonspecific ROS-induced protein oxidation. Moreover, knockdown of another mitochondrial NADPH-producing enzyme, dME3, also results in a CI assembly defect hinting at a critical role for NADPH in CI assembly.

Accordingly, we propose that there are at least two signals that are triggered as a result of dIDH2 disruption to cause the specific order of OXPHOS degeneration observed. The first involves increased ROS levels, but the second is a decrease in the intramitochondrial pool of NADPH. A reduction in the intramitochondrial concentration of NADPH could, in turn, affect CI biogenesis in several ways. First, one of the reactions that occur during the de novo synthesis of [2Fe-2S] clusters requires NADPH. Ferredoxin reductase is reduced by NADPH and then transfers its electrons to Yah1, where they are used for the synthesis of [2Fe-2S] on Isu1 [reviewed in (*49*)]. As this is one of the initiating steps in the synthesis of both [2Fe-2S] and [4Fe-4S] clusters, a slight decrease in ferredoxin reductase reduction by NADPH would severely impair Fe-S cluster biogenesis. The limited amount of intramitochondrial NADPH for Fe-S cluster synthesis is likely the reason why, although forced expression of NDI1 restores biogenesis of an initiating Q-module subcomplex containing NDUFS3, it failed to fully ameliorate the biogenesis defect of the matrix domain that contains eight Fe-S clusters. Second, NADPH is a crucial electron source for several reductive biosynthesis reactions, such as fatty acids, amino acids, nucleotides, and cholesterol synthesis, all of which regulate CI biogenesis either directly or indirectly (*49*–*52*). In view of that, suppressing ROS production or the other ferroptotic signals would be incapable of rescuing the OXPHOS assembly defect because of the paucity of NADPH. This may also explain why raising the dIDH2-KD2 flies on ferrostatin-1 and liproxstatin-1 did not restore OXPHOS assembly to wild-type levels. However, it is also possible that ferroptosis occurs downstream of the OXPHOS assembly defects.

When Fe-S cluster biogenesis is disturbed in the mitochondrial matrix, the amount of free intramitochondrial iron increases. This can generate even more damaging species of ROS in the Fenton reaction and drive lipid peroxidation, ultimately resulting in ferroptosis. Although ferroptotic cell death has not been described in *Drosophila* flight muscles, several lines of evidence lead us to the conclusion that ferroptosis is activated in dIDH2-KD2 flight muscles. First, although the life span of dIDH2-KD2 flies is severely reduced, with most dying within 10 days of eclosure at 25°C, caspase activity in the flight muscles of dIDH2-KD2 flies was less than what is found in wild-type flies. This indicates that apoptosis is not the primary cell death mechanism in dIDH2-KD2 flies. Second, the ferroptotic markers of ROS, lipid peroxidation, and labile iron are all increased in the mitochondrion of dIDH2-KD2 flies relative to wild-type controls. Third, by means of transmission electron micrographs, we observed the hallmarks of ferroptosis of preserved nuclear integrity and shrunken mitochondria in dIDH2-KD2 flight muscles. Crucially, raising the dIDH2-KD2 flies on two ferroptosis inhibitors—ferrostatin-1 and liproxstatin-1—potently rescued their early lethality. When considered together, we conclude that ferroptosis is induced in the flight muscles of dIDH2-KD2 flies.

Our combined proteomics and RNA-seq data indicate that, when dIDH2 is knocked down, it leads to the activation of a robust compensatory stress signaling response that effectively buffers the flies against cell death. As a case in point, extramitochondrial cytochrome c pools were similar between wild-type and dIDH2-KD1 flight muscles, indicating that cytochrome c is not released into the cytosol to cause apoptosis (Fig. 8E). Our proteomic data revealed that several genes that regulate coenzyme Q biosynthesis were up-regulated in dIDH2-KD1 samples. This result is consistent with the induction of a robust adaptive response aimed at maintaining electron transfer through a suboptimal electron transport chain. Most notably, proteins that regulate the UPR^mt^ and other aspects of mitochondrial protein homeostasis were strongly up-regulated in mitochondria from dIDH2-KD1 and dIDH2-KD2 flight muscles. This may be one of the reasons why, despite the induction of ROS in dIDH2-KD1 flies, neither their survival nor locomotory activity was significantly impaired during the first 2 weeks after eclosure.

In summary, we have identified a proferroptotic signaling network activated as a result of disrupting NADPH biosynthesis in the mitochondrion that results in shrunken mitochondria and is associated with impaired OXPHOS assembly. This ferroptotic signaling network involves the activation of several stress-responsive kinases and is counteracted by a robust adaptive response that allows the flies to withstand the proferroptotic cues. While a growing number of reports have shown that ferroptosis is implicated in multiple diseases such as cancer, neurodegeneration, and ischemia/reperfusion injury in the heart, its role in skeletal muscle atrophy or sarcopenia is less clear. Nevertheless, given that skeletal muscles contain approximately 15% of the body’s total iron and an age-dependent iron overload has been observed in skeletal muscles of rats, ferroptosis likely plays a role in muscle aging (*14*, *15*). We anticipate that future studies aimed at thoroughly defining the robust compensatory stress response induced in association with the proferroptotic phenotype in dIDH2-KD1 and dIDH2-KD2 flight muscles, as well as the various phosphorylation events, using the antibodies generated (table S8) will be instrumental in unraveling additional mechanisms by which ferroptosis can be suppressed or induced.

## MATERIALS AND METHODS

### *Drosophila* stocks and genetics

*Drosophila* stocks were reared at 25°C in vials containing agar, molasses, yeast, and cornmeal medium supplemented with propionic acid and methylparaben in humidified incubators (Forma environmental chambers) on a 12-hour:12-hour dark:light cycle. Most of the transgenic stocks used were from the Bloomington *Drosophila* Stock Center (BDSC). The *Drosophila* ortholog of GPX4 (dGPX4) was originally described as GTPx-1 (*53*).

The following transgenic stocks were used: [*y w; Dmef2-Gal4* (*24*)], [w^1118^; P{UAS-eGFP}34/TM3, Sb^1^ (BDSC)], [w^1^; P{UAS-Sod1.A}B36 (BDSC)], [w^1^; P{UAS-Sod2.M}UM83 (BDSC)], [w^1^; P{UAS-Cat.A}2 (BDSC)], [w^1118^; UAS-dGPX4 (*53*)], and [w^1118^; UAS-NDI1 (*36*)]. RNAi stocks for disrupting dSod1/CG11793 were [w^*^; P{UAS-Sod1-IR}4 (BDSC)], [w^*^; P{UAS-Sod1-IR}F103/SM5 (BDSC)], [y^1^ v^1^; P{TRIP.JF03321}attP2 (BDSC)], [y^1^ sc* v^1^ sev^21^; P{TRIP.HMS00698}attP2 (BDSC)], [y^1^ sc* v^1^ sev^21^; P{TRIP.HMS01291}attP2 (BDSC)], and [y^1^ sc* v^1^ sev^21^; P{TRIP.GL01016}attP40 (BDSC)] and were referred to as dSod1^-RNAi-1^ to dSod1^-RNAi-6^, respectively. RNAi stocks for disrupting dSod2/CG8905 were [w^1^; P{UAS-Sod2.dsRNA.K}15/SM5 (BDSC)], [y^1^ sc* v^1^ sev^21^; P{TRIP.HMS00499}attP2 (BDSC)], [y^1^ sc* v^1^ sev^21^; P{TRIP.HMS00783}attP2 (BDSC)], and [y^1^ sc* v^1^ sev^21^; P{TRIP.GL01015}attP40 (BDSC)] and were referred to as dSod2^-RNAi-1^ to dSod2^-RNAi-4^, respectively.

The following transgenic RNAi stocks were also used: dCat/CG6871 [y^1^ sc* v^1^ sev^21^; P{TRIP.HMS00990}attP2 (BDSC)], dME-3/CG5889 [y^1^ sc* v^1^ sev^21^; P{TRIP.HMC04802}attP40 (BDSC)], dPRDX2/CG1633 [y^1^ sc* v^1^ sev^21^; P{TRIP.HMS00501}attP2 (BDSC)], dPRDX3/CG5826 [y^1^v^1^; P{TRIP.HMJ22845}attP40 (BDSC)], dPRDX4/CG1274 [y^1^ sc* v^1^ sev^21^; P{TRIP.HMC04351}attP40 (BDSC)], dPRDX5/CG7217 [y^1^ sc* v^1^ sev^21^; P{TRIP.HMC05872}attP40 (BDSC)], dPRDX6-1/CG3083 [y^1^ sc* v^1^ sev^21^; P{TRIP.HMS05780}attP40/CyO (BDSC)], dPRDX6-2/CG12405 [y^1^v^1^; P{TRIP.HMJ22929}attP40 (BDSC)], dPRDX6-3/CG11765 [y^1^ sc* v^1^ sev^21^; P{TRIP.GL00617}attP40 (BDSC)], dGSTT2/CG10045 [y^1^ sc* v^1^ sev^21^; P{TRIP.GL01039}attP2/TM3, Sb^1^ (BDSC)], dGSTT4/CG12242 [y^1^ sc* v^1^ sev^21^; P{TRIP.HMS02534}attP40 (BDSC)], dHPGDS/CG8938 [y^1^v^1^; P{TRIP.HM05063}attP2 (BDSC)], d*TXNRD2/CG2151* [y^1^v^1^; P{TRIP.HMJ21198}attP40 (BDSC)], and dGPX4/CG12013 [y^1^ sc* v^1^ sev^21^; P{TRIP.HMS00890}attP2 (BDSC)]. The transgenic RNAi stocks for disrupting dIDH2/CG7176 were [w^1118^; P{GD6588}v42916], used in dIDH2-KD1; [P{KK107379}VIE-260B], used in dIDH2-KD2; and [w^1118^; P{GD6588}v42915], used in dIDH2-KD3. All three dIDH2-targeting transgenic RNAi strains were from the Vienna *Drosophila* Resource Center (VDRC). [y^1^ v^1^; P{TRIP.JF01989}attP2 (BDSC)], [y^1^v^1^; P{TRIP.JF02173}attP2 (BDSC)], [y^1^ sc* v^1^ sev^21^; P{TRIP.HMS00935}attP2 (BDSC)], [y^1^v^1^; P{TRIP.HMJ23254}attP2 (BDSC)], and [y^1^ sc* v^1^ sev^21^; P{TRIP.HMS05925}attP40 (BDSC)] targeting dSod2, dCat, dPRDX2, dPRDX6-1, and dGSTT2, respectively, were also tested for OXPHOS defects when crossed with Dmef2-Gal4 but were lethal.

### Locomotory activity

Locomotory activity was assessed in two different ways at 25°C. Locomotory activity was first assessed using a climbing ability assay. In this assay, 25 adult male flies were placed in vials containing fly food. Subsequently, the vials were tapped gently to allow the flies to settle at the bottom. The number of flies that climbed beyond the midpoint of the vial within 15 s was noted and recorded. The experiment was performed in triplicate.

Locomotory activity was also assessed using the *Drosophila* activity monitor (TriKinetics). Specifically, eight adult male flies were placed in the *Drosophila* activity monitor, and spontaneous movements were recorded continuously for 288 hours on a 12-hour:12-hour dark:light cycle.

### Purification of mitochondria

Mitochondrial purification was performed essentially as described previously (*54*), following procedures initially described by Rera *et al.* (*18*). Fly thoraces were quickly dissected and gently crushed with a dounce homogenizer (10 strokes) in 500 μl of a prechilled mitochondrial isolation buffer [250 mM sucrose, 0.15 mM MgCl_2_, and 10 mM tris-HCl (pH 7.4)] supplemented with Halt Protease inhibitors (Pierce). Tissue homogenates were centrifuged twice at 500*g* for 5 min at 4°C to remove the cuticle and other insoluble material. Subsequently, the supernatant was recovered and centrifuged at 5000*g* for 5 min at 4°C to obtain the mitochondria-enriched pellet, which was washed twice in the mitochondrial isolation buffer and stored at −80°C until further processing.

### Blue native polyacrylamide gel electrophoresis

BN-PAGE was performed using NativePAGE gels (Life Technologies) and following the manufacturer’s protocol as described previously (*54*, *55*). The digitonin:protein ratio used was 10 g of digitonin:3 g of protein.

### Silver staining

Silver staining of blue native gels was performed with a SilverXpress staining kit (Life Technologies) as described previously (*54*, *55*), following the manufacturer’s instructions.

### In-gel CI, CII, CIV, and CV activity

In-gel OXPHOS activities were performed as described previously (*54*, *55*). In-gel CI activity was assessed by incubating the native gels in NADH (0.1 mg/ml), nitrotetrazolium blue chloride (NTB; 2.5 mg/ml), and 5 mM tris-HCl (pH 7.4) at 25°C. In-gel CII activity was assessed by incubating the native gels in 20 mM sodium succinate, 0.2 mM phenazine methosulfate, NTB (2.5 mg/ml), and 5 mM tris-HCl (pH 7.4) at 25°C. In-gel CIV activity was assessed by incubating the native gels in 50 mM sodium phosphate (pH 7.2), 0.05% 3,3′-diaminobenzidine tetrahydrochloride, and 50 μM horse heart cytochrome c at 25°C. In-gel CV activity was assessed by preincubating the gel in 35 mM tris-base and 0.27 M glycine (pH 8.4) for 3 hours and then subsequently in 35 mM tris-base, 0.27 M glycine (pH 8.4), 14 mM MgSO_4_, 0.2% (w/v) Pb(NO_3_)_2_, and 8 mM adenosine triphosphate (ATP) at 25°C.

### Amplex Red assay for measuring hydrogen peroxide production

The amount of hydrogen peroxide produced was monitored using an Amplex Red hydrogen peroxide assay kit (Molecular Probes), as described previously (*54*). Briefly, 10 fly thoraces were homogenized in prechilled mitochondrial isolation buffer supplemented with Halt Protease inhibitors and centrifuged twice at 500*g* for 5 min at 4°C to remove insoluble material. Subsequently, a serial dilution of the supernatant was added to a reaction buffer containing 100 μM Amplex Red reagent and horseradish peroxidase solution (0.2 U/ml). Fluorescence was measured at an excitation wavelength of 540 nm and detected at 590 nm every 30 s for 30 min at 25°C using a SpectraMax paradigm multimode microplate reader (Molecular Devices). The background fluorescence determined for a blank reaction (no H_2_O_2_) was deducted from each value. Amplex Red activity was normalized to protein concentrations, as determined with a microBCA kit (Thermo Fisher Scientific). Samples were analyzed in triplicate.

### Lipid peroxidation assay

The extent of lipid peroxidation in fly thoraces was assessed using a lipid peroxidation assay kit (MilliporeSigma). Thirty fly thoraces were homogenized in 300 μl of malondialdehyde (MDA) lysis buffer and 3 μl of 100× butylated hydroxytoulene (BHT). Both solvents were supplied with the kit. The samples were subsequently centrifuged at 13,000*g* for 10 min to remove the cuticle and other insoluble material. After protein concentrations were normalized between samples, 200 μl of each sample was aliquoted into a separate Eppendorf tube. Subsequently, 200 μl of perchloric acid was added to each tube, vortexed, and centrifuged at 13,000*g* for 10 min. A total of 200 μl of the recovered supernatant was transferred into a new Eppendorf tube, and 600 μl of the thiobarbituric acid (TBA) solution (supplied with the kit) was added to form the MDA-TBA adduct. The samples were subsequently incubated at 95°C for 60 min and then placed in an ice bath for 10 min. To enhance sensitivity, 300 μl of 1-butanol was added to extract the MDA-TBA adduct from the 800-μl reaction mixture and then centrifuged at 16,000*g* for 3 min at room temperature to separate the layers. The fraction containing 1-butanol (i.e., the top layer) was transferred to another tube. Following evaporation of 1-butanol, the residue containing the MDA-TBA adduct was dissolved in 200 μl of water and then transferred to a 96-well plate. Absorbance of the MDA-TBA adduct was measured at 532 nm, and the amount of MDA present in triplicate samples was assessed using an MDA standard graph.

### Quantification of NADP/NADPH ratio

The NADP/NADPH ratio was measured in fly thoraces with an NADP/NADPH quantification kit (MilliporeSigma). Ten fly thoraces were homogenized in 500 μl of NADP/NADPH extraction buffer (supplied with the kit) and 5 μl of 100× Halt Protease inhibitors. Afterward, the samples were centrifuged at 10,000*g* for 10 min to remove insoluble material. Any residual particulate matter was removed by transferring the extracted NADP/NADPH supernatant into a 10-kDa cutoff spin filter and by centrifuging at 10,000*g* for 20 min at 4°C. The flow-through (extracted samples) was saved for further analyses. To detect NADPH, NADP was decomposed by aliquoting 200 μl of the extracted samples into Eppendorf tubes and heating to 60°C for 30 min in a heating block. Samples were cooled on ice.

Fifty microliters of both the decomposed NADP (measures NADPH only) and regular samples (measures both NADPH and NADP) was aliquoted into 96-well plates, and 100 μl of the reaction mix supplied with the kit was added to each of the standard and sample wells. The samples were mixed thoroughly and incubated for 5 min at 25°C to convert NADP to NADPH. Ten microliters of an NADPH developer (supplied with the kit) was added to each sample and incubated at room temperature for 60 min, after which the absorbance of both samples was measured at 450 nm. The reaction was subsequently stopped by adding 10 μl of stop solution (supplied with the kit) to each well and mixing thoroughly. The background value for the assay was the value obtained for the blank NADPH standard. After correcting for the background value, the amount of NADPH present in the sample was assessed using the NADPH standard curve. Samples were analyzed in triplicate. The NADP/NADPH ratio was obtained by using the following formula: NADP/NADPH ratio = (NADP_total_ − NADPH)/NADPH.

### Measurement of mitochondrial free Fe^2+^ ions

The mitochondrial free ferrous iron was measured using a QuantiChrom iron assay kit (BioAssay). Mitochondria from 40 thoraces were suspended in 300 μl of the medium containing 5 mM PIPES buffer (pH 6.5) and incubated at 37°C for 30 min to produce a hypo-osmotic swelling. The ruptured mitochondrial sample was subsequently centrifuged at 10,000*g* for 5 min, after which the supernatant was recovered. Two hundred microliters of the working reagent (supplied with the kit) was added to 100 μl of the supernatant in a 96-well plate. After incubation for 40 min at room temperature, the absorbance was measured at 590 nm. The background value for the assay (blank) was used together with the varying concentrations of Fe^2+^ to plot the standard curve. After correcting for background values, the amount of Fe^2+^ present in the sample was estimated using the Fe^2+^ standard curve. Samples were analyzed in triplicate.

### Caspase activity assay

Caspase 3 activity was measured by using a caspase 3 fluorimetric assay kit (MilliporeSigma). In this case, 10 fly thoraces were homogenized in 1× phosphate-buffered saline supplemented with Halt Protease inhibitors and centrifuged twice at 500*g* for 5 min at 4°C to remove insoluble material. Two hundred microliters of the reaction mixture (supplied with the kit) containing 250 μM caspase 3 substrate (Ac-DEVD-AMC) was added to each of the standards and samples, thoroughly mixed, and incubated for 60 min at room temperature protected from light. Fluorescence was measured at an excitation wavelength of 360 nm and detected at 460 nm every 30 s for 60 min at 25°C using a SpectraMax microplate reader. After correcting for background values, caspase 3 activity was assessed using the 7-amino-4-methylcoumarin (AMC) standard curve and analyzed in triplicate.

### Ferroptosis rescue

Flies of the appropriate genotype were allowed to lay eggs for 48 hours at 25°C. After the ensuing larvae reached the third instar, 200 μl each of ferrostatin-1 (1 mg/ml) and liproxstatin-1 (1 mg/ml) was added to the fly food, and the larvae were allowed to continue developing in the midst of the drug until they eclosed. The adult flies were reared at a density of 25 flies per vial and maintained at 25°C. Flies were transferred to vials of fresh fly food every other day and scored daily for viability.

### Electron microscopy

Dissected thoraces were fixed in 2.5% glutaraldehyde and 4% paraformaldehyde in 0.1 M cacodylate buffer for 48 hours at 4°C and then postfixed in buffered 1% osmium tetroxide. The samples were subsequently dehydrated in a graded series of acetone and embedded in EMbed 812 resin (Electron Microscopy Sciences). Ninety-nanometer-thin sections were cut on a Leica UC6 ultramicrotome and stained with a saturated solution of uranyl acetate and lead citrate. Images were captured with an AMT (Advanced Microscopy Techniques) XR111 digital camera at 80 kV on a Philips CM12 transmission electron microscope.

### Generation of peptide polyclonal antibodies

Rabbit polyclonal antibodies recognizing various segments of specific target proteins in *Drosophila* were generated by Biomatik using the synthetic peptides listed in table S8.

### Immunoblotting

Immunoblotting was performed as previously described (*17*). In addition to the new rabbit polyclonal antibodies we generated, the following primary antibodies were also used: anti-dNDUFS2 (*17*), anti-NDUFS3 (Abcam, ab14711), anti-dNDUFS5 (*17*), anti-dNDUFS7 (*54*), anti-dNDUFS8 (*54*), anti-dNDUFA8 (*54*), anti-dNDUFA11 (*54*), anti-dNDUFA12 (*54*), anti-dNDUFV1 (*17*), anti-dNDUFV3 (*54*), anti-dNDUFB5 (*17*), anti-dNDUFB6 (*17*), anti-dNDUFB8 (*17*), anti-dND1 (*17*), anti-dND2 (*17*), anti-dND3 (*17*), anti-dND4L (*17*), anti-dND5 (*17*), anti-dND6 (*17*), anti-VDAC (Abcam, ab14734), anti-HSP60 (CST, #4870S), anti–cytochrome c (ab13575), anti–phospho-AMPKα (Thr^172^) [Cell Signaling Technology (CST), #2535], anti-HSP90 (CST, #4874), anti–phospho-eIF2α (Ser^51^) (CST, #3398), anti–phospho-p44/42 MAPK (Erk1/2) (Thr^202^/Tyr^204^) (CST, #4377), anti–phospho-p38 MAPK (Thr^180^/Tyr^182^) (CST, #9215), anti–phospho-SAPK/JNK (Thr^183^/Tyr^185^) (CST, #4668), anti–phospho-SEK1/MKK4 (Ser^257^/Thr^261^) (CST, #9156), and anti-ATPsynβ (Life Technologies, A21351). Secondary antibodies used were goat anti-rabbit horseradish peroxidase (PI31460 from Pierce) and goat anti-mouse horseradish peroxidase (PI31430 from Pierce).

### In-gel digestion and MS

In-gel digestion and MS were performed as described previously (*54*). OXPHOS complexes from mitochondrial preparations from *Dmef2-Gal4/w^1118^* and *Dmef2-Gal4/UAS-dIDH2^-RNAi-1^* flies were separated on blue native gels. Each genotype was run in triplicate. Following separation of the OXPHOS complexes, the gel was incubated in a fixative consisting of 50% methanol, 10% acetic acid, and 100 mM ammonium acetate for 30 min. Afterward, the gel was washed twice with ultrapure water. Six gel slices corresponding to the holoenzyme and five slices between the region where CIV and CII migrate were excised for each genotype. The excised gel slices were further diced into smaller pieces, placed in Eppendorf tubes, and destained in a gel destaining buffer (8% acetic acid). In-gel trypsin digestion was performed essentially as described previously (*56*). In brief, 100 μl of 25 mM dithiothreitol and 100 μl of 50 mM iodoacetamide were used for protein reduction and alkylation, respectively, followed by digestion with 0.5 μg of trypsin at 37°C for 16 hours. The tryptic peptides were extracted and desalted using a C_18_ cartridge, followed by liquid chromatography–MS/MS (LC-MS/MS) analysis on an Orbitrap Fusion Lumos Tribrid mass spectrometer (Thermo Fisher Scientific). The peptides were separated on a C_18_ nanocolumn (75 μm by 50 cm, 2 μm, 100 Å) with a 2-hour linear gradient consisting of solvent A [2% acetonitrile in 0.1% formic acid (FA)] and solvent B (85% acetonitrile in 0.1% FA). The eluted peptides were directly introduced to the MS via a nanospray flex ion source. The MS spectra were acquired in the positive mode with a spray voltage of 2 kV. The temperature of the ion transfer tube was 275°C. MS scan range was between *m/z* (mass/charge ratio) 375 and 1500 with a 120,000 (full width at half maximum) resolution in the Orbitrap MS. The peptides with charge states between 2 and 7 were selected for MS/MS analysis. Higher-energy collisional dissociation was used for peptide fragmentation, with a collision energy of 30%.

### Protein identification and quantification

The MS/MS spectra were searched against the UniProt *Drosophila* database (21,107 entries, downloaded on 12 November 2020) using the Sequest search engine through the Proteome Discoverer (version 2.4) platform. The mass tolerance was 10 parts per million for MS and 0.6 Da for MS/MS. Methionine oxidation and N-terminal acetylation were set as variable modifications, and cysteine carbamidomethylation was set as a fixed modification. The false discovery rate accepted for the identification of both proteins and peptides was less than 1%. Relative protein quantitation was calculated on the basis of the spectral counting method (*32*, *57*). To circumvent the problem with large spectrum count (SC) ratios from small SCs in the SC ratio denominators, we arbitrarily added two SCs for each protein before calculating the protein quantification ratios.

### RNA sequencing

We dissected 10 thoraces of each genotype in triplicate and isolated total RNA with TRIzol reagent (Thermo Fisher Scientific) following the manufacturer’s instructions. Additional RNA processing, sequencing reactions, and bioinformatic analyses were performed at GENEWIZ LLC, which has now become Azenta Life Sciences US Inc., as follows.

### Library preparation with polyA selection and HiSeq sequencing

RNA samples were quantified using a Quibit 2.0 fluorometer (Life Technologies), and RNA integrity was checked using Agilent TapeStation 4200 (Agilent Technologies). RNA-seq libraries were prepared using a NEBNext Ultra II RNA library preparation kit for Illumina following the manufacturer’s instructions (NEB). Briefly, mRNAs were first enriched with oligo(dT) beads. Next, the enriched mRNAs were fragmented for 15 min at 94°C. First- and second-strand cDNAs were then synthesized. The cDNA fragments were end-repaired and adenylated at their 3′ ends, and universal adapters were ligated to the cDNA fragments, followed by index addition and library enrichment by limited-cycle PCR. The sequencing libraries were validated on the Agilent TapeStation and quantified with a Quibit 2.0 fluorometer, as well as by quantitative RT-PCR (Kapa Biosystems).

The sequencing libraries were clustered on two flow cell lanes. After clustering, the flow cell was loaded on the Illumina HiSeq instrument (4000 or equivalent) according to the manufacturer’s instructions. The samples were sequenced using a 2 × 150–base pair paired end configuration. Image analysis and base calling were conducted by the HiSeq Control Software. Raw sequence data (.bcl files) generated from the Illumina HiSeq were converted into fastq files and demultiplexed using Illumina’s bcl2fastq software (version 2.17). One mismatch was allowed for index sequence identification.

### Bioinformatics

After investigating the quality of the raw data, sequence reads were trimmed to remove possible adapter sequences and nucleotides with poor quality using Trimmomatic v0.36. The trimmed reads were mapped to the *Drosophila melanogaster* (BDGP6) reference genome available on ENSEMBL using the STAR aligner v.2.5.2b. BAM files were generated as a result of this step. Unique gene hit counts were calculated by using feature counts from the subread package v.1.5.2. Only unique reads that fell within exon regions were counted. After extraction of gene hit counts, the gene hit counts table was used for downstream differential expression analysis. Using DESeq2, a comparison of gene expression between wild-type samples and samples where dIDH2 had been knocked down was performed. The Wald test was used to generate *P* values and log_2_ fold changes. Genes with adjusted *P* values of <0.05 and absolute log_2_ fold changes of >0.6 were called as differentially expressed. Gene ontology (GO) analysis was performed on the statistically significant set of genes by implementing the software GeneSCF. The GO annotation for FlyBase (*D. melanogaster*) GO list was used to cluster the set of genes based on their biological processes, after which their statistical significance was determined.

### Quantitative real-time PCR

Quantitative real-time PCR was performed as described previously (*17*). Total RNA was isolated from thoraxes using TRIzol Reagent (Thermo Fisher Scientific) and, following the elimination of genomic DNA, reverse-transcribed using an iScript cDNA Synthesis kit (Bio-Rad). The PCR reaction was performed using Bio-Rad’s iQ SYBR Green Supermix.

The following primer sequences, listed 5′ to 3′, were used: Hsp70Ab, AGATTATCGCCAACGACCAG (forward) and TCATGTCCTCTGCGATCTTG (reverse); Impl3, CGTTTGGTCTGGAGTGAACA (forward) and CCCAGGAGGTGTATCCCTTT (reverse); Alr, CGCGACATGAAGACCTTTTT (forward) and ACCTTGGTGCAATCGAAGAG (reverse); Qtzl, GAGACAGGTGCGATTCAACA (forward) and CGAAGCGTACGAACCAGATT (reverse); Fdx1, AGGACGACCTGCTGGATATG (forward) and TTAATGTGGCTTGGGCTTGT (reverse); PHGPx, GATACCCATGGCAACGATGT (forward) and CATCTGGGACCCAAACTGAT (reverse); Fer1HCH, CCAGGCCTATGGAGATTTCA (forward) and CTTCATGTCCACCCAGTCCT (reverse); Nfs1, ATCGCAGTAAAGGGTGTTGC (forward) and GAGGTCTCCGATGTGATGGT (reverse); and Bcn92, AGCTGCCCTCGTACAACTTC (forward) and GAATACAGGTGGCCGATGAT (reverse).

### Statistics

For statistical analyses and graphical display of data, the GraphPad Prism 9 software was used. To evaluate whether differences were observed in data from two groups, significance was expressed as *P* values based on Student’s *t* test for unpaired two-tailed samples. For comparisons involving more than two groups and where some experimental groups were compared with each other, one-way analysis of variance (ANOVA) with Tukey’s multiple comparisons test was performed when parametric tests were deemed appropriate. For assessments where two or more experimental groups were compared against a single control group, one-way ANOVA followed by the Dunnett’s test was used. When evaluating grouped analyses with multiple comparisons and each comparison was considered independent of others, the two-way ANOVA with Sidak multiple comparisons test was performed. The fold change shown in all graphs refers to the mean ± SEM, and significance was depicted as *P < 0.05, **P < 0.01, ***P < 0.001, and ****P < 0.0001.

## Notes

View/request a protocol for this paper from *Bio-protocol*.
