# Supplementary material for: IDH2-mediated regulation of the biogenesis of the oxidative phosphorylation system
Source: Sci Adv. 2022 May 11;8(19):eabl8716. doi: 10.1126/sciadv.abl8716 (PMC9094667; doi:10.1126/sciadv.abl8716)
Supplement: Supplementary file 1 — Figs. S1 to S18 [file sciadv.abl8716_sm.pdf]

Science Advances
AAAS

Supplementary Materials for

IDH2-mediated regulation of the biogenesis of the oxidative phosphorylation system

Anjaneyulu Murari, Naga S. V. Goparaju, Shauna-Kay Rhooms, Kaniz F. B. Hossain, Felix G. Liang, Christian J. Garcia, Cindy Osei, Tong Liu, Hong Li, Richard N. Kitsis, Rajesh Patel, Edward Owusu-Ansah*

*Corresponding author. Email: eo2364@cumc.columbia.edu

Published 11 May 2022, *Sci. Adv.* **8**, eabl8716 (2022)
DOI: 10.1126/sciadv.abl8716

The PDF file includes:

Figs. S1 to S18
Legends for tables S1 to S8

Other Supplementary Material for this manuscript includes the following:

Tables S1 to S8

Figure S1: Replicates of immunoblots shown in Figures 1E-1G

The figure legend is the same as what is described for figure panels 1E-1G.

Figure S2: Additional replicates of immunoblots shown in Figures 1E-1G

The figure legend is the same as what is described for figure panels 1E-1G.

Figure S3: Quantification of immunoblots shown in Figures 1E-1G

The antibodies quantified for 1E-1G are listed above each graph. In all instances except the 1F panels, p values are based on one-way ANOVAs followed by the Dunnett's test. In the 1F panels, p values are based on the student's t-test for unpaired two-tailed samples. The fold change shown refers to the mean ± s.e.m (standard error of the mean); and n.s. denotes p > 0.05, * = p<0.05, ** = p<0.01 and *** = p<0.001. The number of replicates (n) = 3 biological replicates, with 15 flies per replicate.

Figure S4: NADPH or ferroptosis inhibitors fail to rescue the OXPHOS assembly defects observed when dIDH2 is knocked down.

(A-F) Mitochondrial preparations from thoraces isolated from Dmef2-Gal4/w1118 (wild type) and dIDH2-KD1 flies raised on a diet supplemented with 5mM and 10mM NADPH 4 days after eclosure. The mitochondrial preparations were analyzed by BN-PAGE, followed by immunoblotting with the antibodies indicated. The blots were imaged following a short exposure to detect the holoenzyme and supercomplexes, after which the region corresponding to the holoenzyme and supercomplexes was cut off, and the rest of the blot re-imaged after a longer exposure to detect the assembly intermediates (denoted as *). The # refers to the initiating AI of the Q-module. The antibodies used were anti-NDUFS3 which detects dNDUFS3 (A), anti-dNDUFS7 (B), anti-dNDUFS8 (C), anti-dNDUFA9 (D), anti-dND1 (E), and anti-dND3 (F). Anti-ATP synthase, subunit B (ATP5F1B) which detects the *Drosophila* ortholog, dATP-Synβ, was used as a loading control.

(G-J) Mitochondrial preparations from thoraces isolated from Dmef2-Gal4/w1118 (wild type) and dIDH2-KD2 flies raised on a diet supplemented with 1mg/ml each of ferrostatin-1 and liproxstatin-1 (FL-1) 4 days after eclosure. The antibodies used were anti-NDUFS3 which detects dNDUFS3

(G), anti-dNDUFA9 (H), anti-dNDUFV3 (I) and anti-dNDUFB5 (J). Anti-ATP synthase, subunit B (ATP5F1B) which detects the *Drosophila* ortholog, dATP-Synβ, was used as a loading control.

Figure S5: Replicates of immunoblots shown in Figure 4

The figure legend is the same as what is described for Figure 4.

Figure S6: Additional replicates of immunoblots shown in Figure 4

The figure legend is the same as what is described for Figure 4.

Figure S7: Quantification of immunoblots shown in Figure 4

The antibody quantified for each figure panel is listed above each graph. In all instances, p values are based on one-way ANOVAs followed by the Dunnett's test. The fold change shown refers to the mean $\pm$ s.e.m (standard error of the mean); and n.s. denotes $p > 0.05$, * = $p<0.05$, ** = $p<0.01$ and *** = $p<0.001$. The number of replicates (n) = 3 biological replicates, with 40 flies per replicate.

Figure S8: Replicates of immunoblots shown in Figure 5

The figure legend is the same as what is described for Figure 5.

Figure S9: Additional replicates of immunoblots shown in Figure 5

The figure legend is the same as what is described for Figure 5.

Figure S10: Quantification of immunoblots shown in Figure 5

The antibody quantified for each figure panel is listed above each graph. In all instances, p values are based on one-way ANOVAs followed by the Dunnett's test. The fold change shown refers to the mean $\pm$ s.e.m (standard error of the mean); and n.s. denotes $p > 0.05$, * = $p<0.05$, ** = $p<0.01$ and *** = $p<0.001$. The number of replicates (n) = 3 biological replicates, with 40 flies per replicate.

Figure S11: Replicates of immunoblots shown in Figure 8

The figure legend is the same as what is described for Figure 8.

Figure S12: Additional replicates of immunoblots shown in Figure 8

The figure legend is the same as what is described for Figure 8.

Figure S13: Quantification of immunoblots shown in Figures 8A-8C

The antibodies quantified for figure panels are listed above each graph. In 8C, p values are based on one-way ANOVAs followed by Dunnett's multiple comparisons test. In 8A and 8B, one-way ANOVAs followed by Tukey's multiple comparisons test was used. The fold change shown refers to the mean ± s.e.m (standard error of the mean); and n.s. denotes $p > 0.05$, * = $p<0.05$, ** = $p<0.01$ and *** = $p<0.001$. The number of replicates (n) = 3 biological replicates, with 15 flies per replicate.

Figure S14: Quantification of immunoblots shown in Figures 8D and 8E

The antibodies quantified for the figure panels are listed above each graph. Due to recurrent phosphorylation-dephosphorylation oscillations, we quantified each kinase experiment separately in Figure 8D. The p values in 8E are based on one-way ANOVAs followed by Dunnett's multiple comparisons test. The fold change shown refers to the mean ± s.e.m (standard error of the mean); and n.s. denotes $p > 0.05$, * = $p<0.05$, ** = $p<0.01$ and *** = $p<0.001$. The number of replicates (n) = 3 biological replicates, with 15 flies per replicate.

Figure S15: Quantification of immunoblots shown in Figure 8F

The antibody quantified for each figure panel is listed above each graph. Due to recurrent phosphorylation-dephosphorylation oscillations, we quantified each kinase experiment separately. For HSP60 and cytochrome C, one-way ANOVAs followed by Tukey's multiple comparisons test was used. The fold change shown refers to the mean ± s.e.m (standard error of the mean); and n.s. denotes $p > 0.05$, * = $p<0.05$, ** = $p<0.01$ and *** = $p<0.001$. The number of replicates (n) = 3 biological replicates, with 15 flies per replicate.

Figure S16: Replicates of immunoblots shown in Figure 9

The figure legend is the same as what is described for Figure 9.

Figure S17: Additional replicates of immunoblots shown in Figure 9

The figure legend is the same as what is described for Figure 9.

Figure S18: Quantification of immunoblots shown in Figure 9

The antibodies quantified for the figure panels are listed above each graph. In all instances, p values are based on the student's t-test for unpaired two-tailed samples. The fold change shown refers to the mean ± s.e.m (standard error of the mean); and n.s. denotes $p > 0.05$, * = $p<0.05$, ** = $p<0.01$ and *** = $p<0.001$. The number of replicates (n) = 3 biological replicates, with 40 flies per replicate.

Table S1: Comparison of identified proteins and their relative quantitation among four A samples using the spectra counting method (sorted by protein name)

Table S2: Comparison of identified proteins and their relative quantitation among four B samples using the spectra counting method (sorted by protein name)

Table S3: Comparison of identified proteins and their relative quantitation among four C samples using the spectra counting method (sorted by protein name)

Table S4: Comparison of identified proteins and their relative quantitation among four D samples using the spectra counting method (sorted by protein name)

Table S5: Comparison of identified proteins and their relative quantitation among four E samples using the spectra counting method (sorted by protein name)

Table S6: Comparison of identified proteins and their relative quantitation among four F samples using the spectra counting method (sorted by protein name)

Table S7: RNA-seq profile when dIDH2 is disrupted in flight muscles

Table S8: A list of the peptide antigens and their target proteins.

FIGURE S1

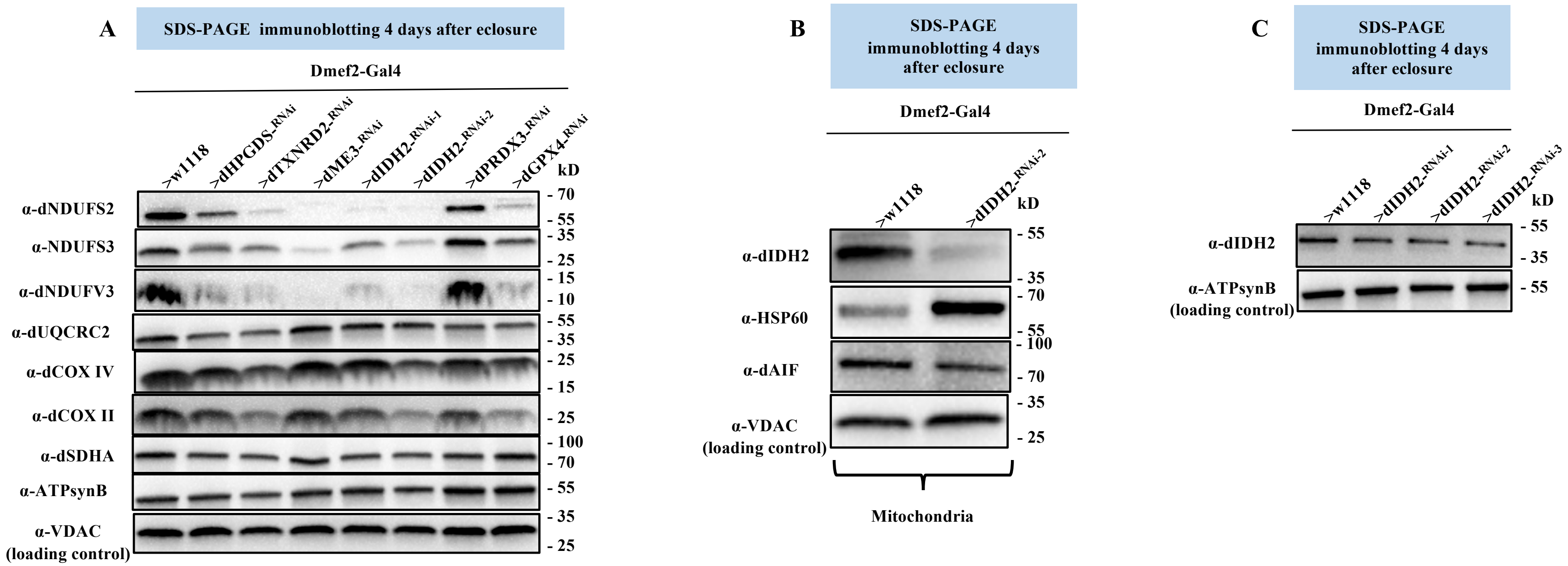

A
SDS-PAGE immunoblotting 4 days after eclosure
Dmef2-Gal4
>w1118
>dHPGDS-RNAi
>dTXNRD2-RNAi
>dME3-RNAi
>dIDH2-RNAi-1
>dIDH2-RNAi-2
>dPRDX3-RNAi
>dGPX4-RNAi
kD
α-dNDUFS2
α-NDUFS3
α-dNDUFV3
α-dUQCRC2
α-dCOX IV
α-dCOX II
α-dSDHA
α-ATPsynB
α-VDAC
(loading control)
- 70
- 55
- 35
- 25
- 15
- 10
- 55
- 35
- 25
- 15
- 25
- 100
- 70
- 55
- 35
- 25
B
SDS-PAGE immunoblotting 4 days after eclosure
Dmef2-Gal4
>w1118
>dIDH2-RNAi-2
kD
α-dIDH2
α-HSP60
α-dAIF
α-VDAC
(loading control)
- 55
- 35
- 70
- 55
- 100
- 70
- 35
- 25
Mitochondria
C
SDS-PAGE immunoblotting 4 days after eclosure
Dmef2-Gal4
>w1118
>dIDH2-RNAi-1
>dIDH2-RNAi-2
>dIDH2-RNAi-3
kD
α-dIDH2
α-ATPsynB
(loading control)
- 55
- 35
- 55

FIGURE S2

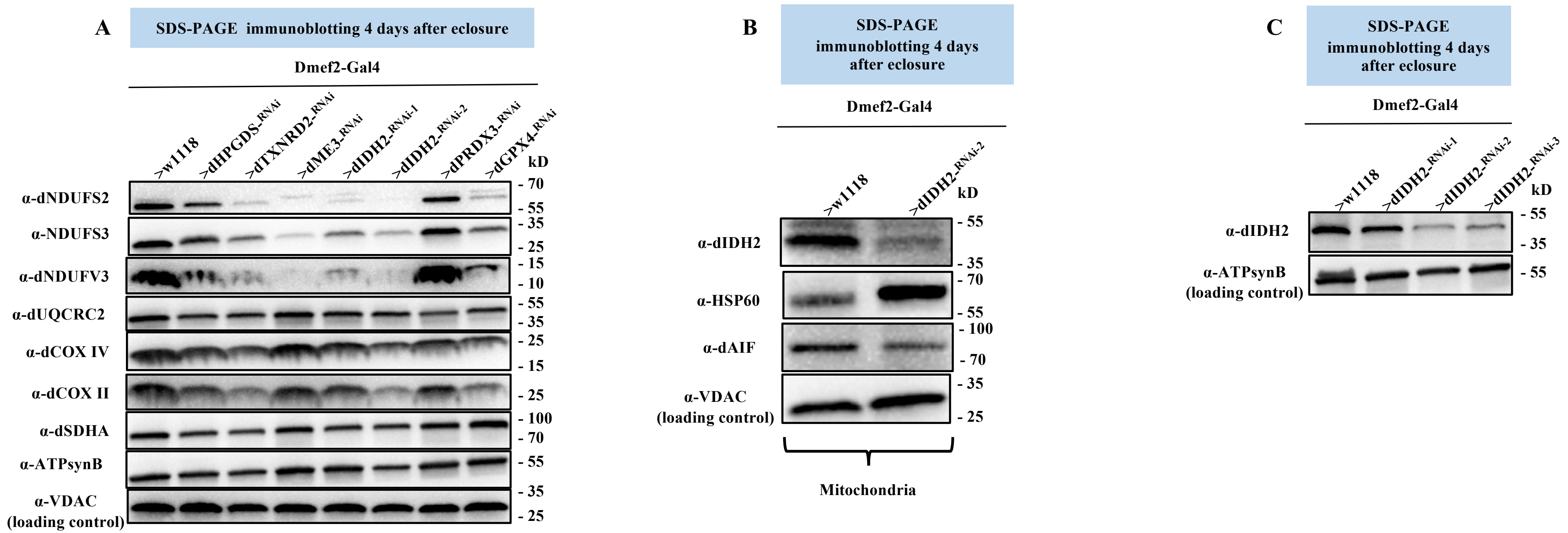
A
SDS-PAGE immunoblotting 4 days after eclosure
Dmef2-Gal4
>w1118
>dHPGDS-RNAi
>dTXNRD2-RNAi
>dME3-RNAi
>dIDH2-RNAi-1
>dIDH2-RNAi-2
>dPRDX3-RNAi
>dGPX4-RNAi
kD
α-dNDUFS2
α-NDUFS3
α-dNDUFV3
α-dUQCRC2
α-dCOX IV
α-dCOX II
α-dSDHA
α-ATPsynB
α-VDAC
(loading control)
- 70
- 55
- 35
- 25
- 15
- 10
- 55
- 35
- 25
- 15
- 25
- 100
- 70
- 55
- 35
- 25
B
SDS-PAGE
immunoblotting 4 days
after eclosure
Dmef2-Gal4
>w1118
>dIDH2-RNAi-2
kD
α-dIDH2
α-HSP60
α-dAIF
α-VDAC
(loading control)
- 55
- 35
- 70
- 55
- 100
- 70
- 35
- 25
Mitochondria
C
SDS-PAGE
immunoblotting 4 days
after eclosure
Dmef2-Gal4
>w1118
>dIDH2-RNAi-1
>dIDH2-RNAi-2
>dIDH2-RNAi-3
kD
α-dIDH2
α-ATPsynB
(loading control)
- 55
- 35
- 55

FIGURE S3

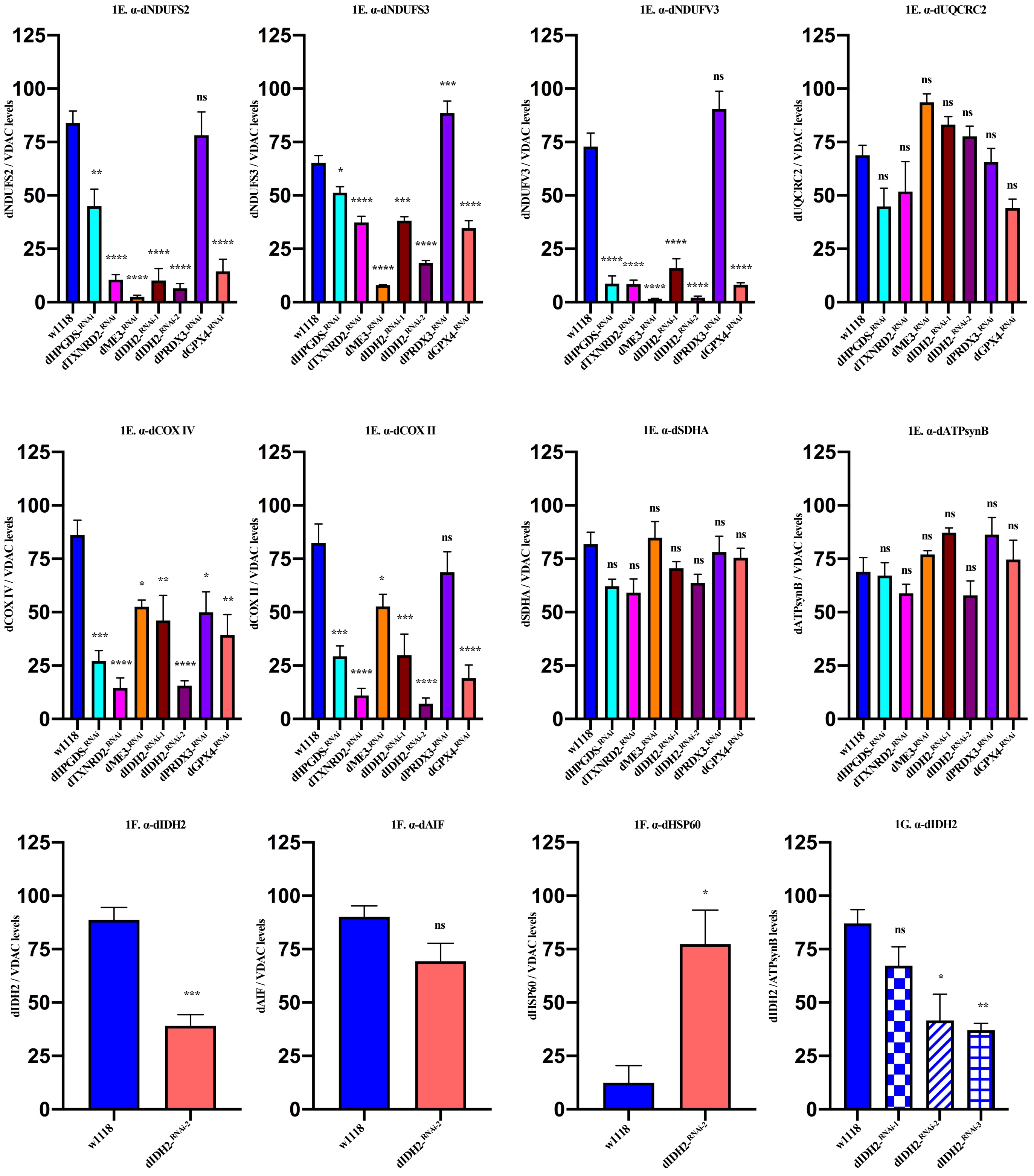
1E. α-dNDUFS2
dNDUFS2 / VDAC levels
1E. α-dNDUFS3
dNDUFS3 / VDAC levels
1E. α-dNDUFV3
dNDUFV3 / VDAC levels
1E. α-dUQCRC2
dUQCRC2 / VDAC levels
1E. α-dCOX IV
dCOX IV / VDAC levels
1E. α-dCOX II
dCOX II / VDAC levels
1E. α-dSDHA
dSDHA / VDAC levels
1E. α-dATPsynB
dATPsynB / VDAC levels
w1118
dHPGDS-RNAi
dTXNRD2-RNAi
dME3-RNAi
dIDH2-RNAi-1
dIDH2-RNAi-2
dPRDX3-RNAi
dGPX4-RNAi
1F. α-dIDH2
dIDH2 / VDAC levels
1F. α-dAIF
dAIF / VDAC levels
1F. α-dHSP60
dHSP60 / VDAC levels
1G. α-dIDH2
dIDH2 /ATPsynB levels
dIDH2-RNAi-1
dIDH2-RNAi-2
dIDH2-RNAi-3

FIGURE S4

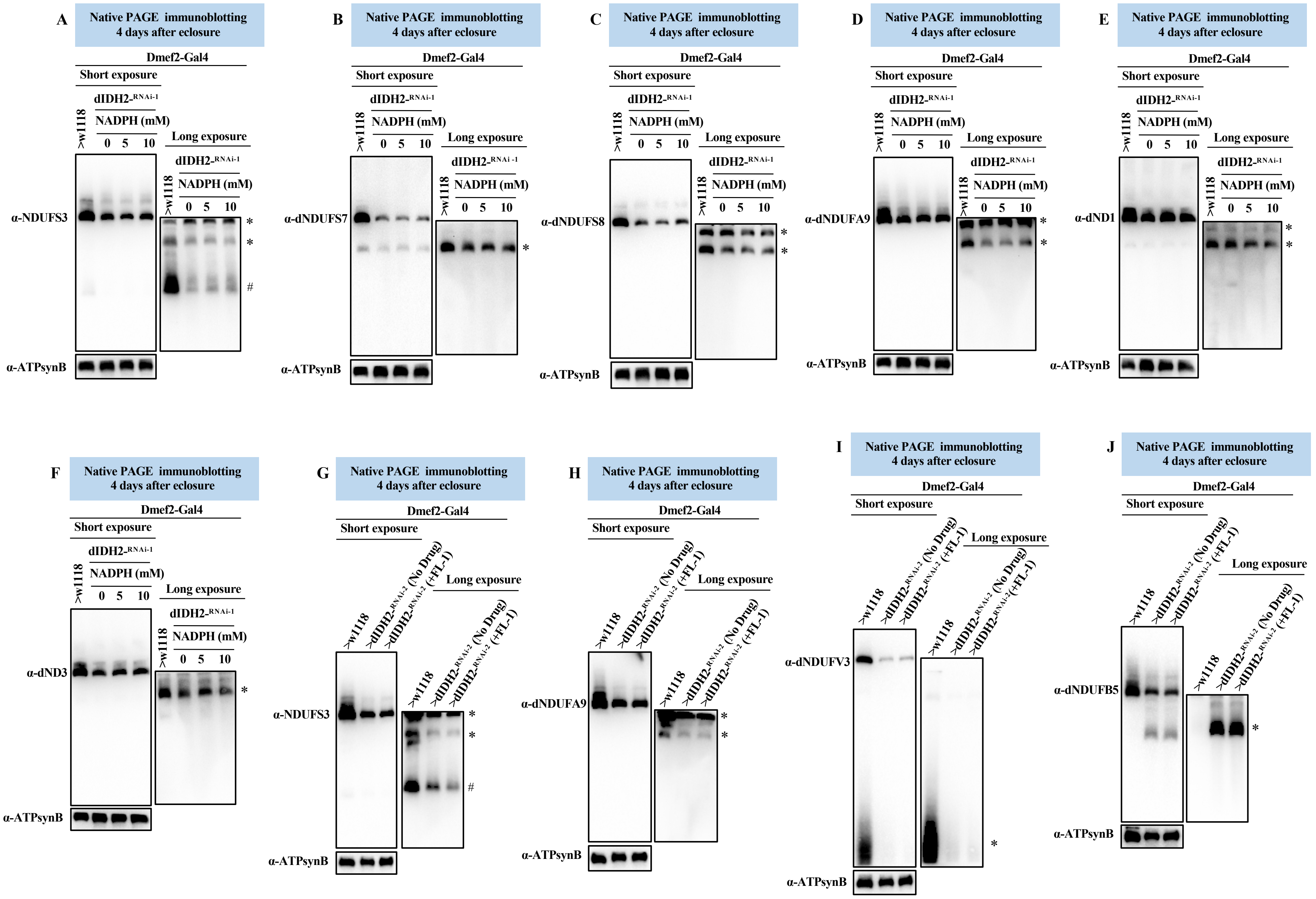

A
B
C
D
E
Native PAGE immunoblotting
4 days after eclosure
Dmef2-Gal4
Short exposure
Long exposure
dIDH2-RNAi-1
NADPH (mM)
>w1118
0 5 10
α-NDUFS3
α-dNDUFS7
α-dNDUFS8
α-dNDUFA9
α-dND1
α-ATPsynB
F
G
H
I
J
α-dND3
α-NDUFS3
α-dNDUFA9
α-dNDUFV3
α-dNDUFB5
>dIDH2-RNAi-2 (No Drug)
>dIDH2-RNAi-2 (+FL-1)

FIGURE S5

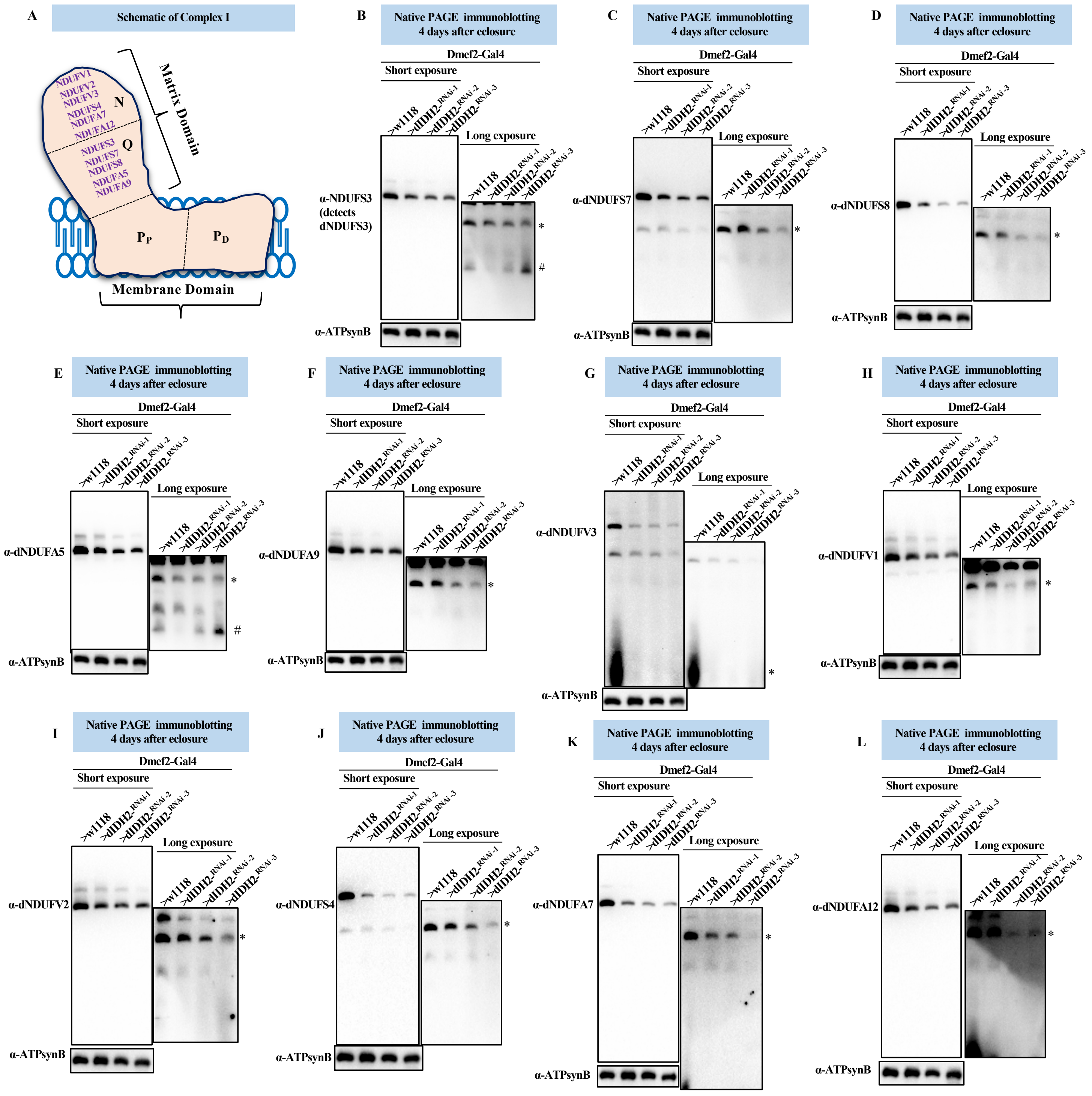
A
Schematic of Complex I
NDUFV1
NDUFV2
NDUFV3
NDUFS4
NDUFA7
NDUFA12
N
Q
NDUFS3
NDUFS7
NDUFS8
NDUFA5
NDUFA9
Matrix Domain
PP
PD
Membrane Domain
B
C
D
E
F
G
H
I
J
K
L
Native PAGE immunoblotting
4 days after eclosure
Dmef2-Gal4
Short exposure
Long exposure
w1118
dIDH2RNAi-1
dIDH2RNAi-2
dIDH2RNAi-3
α-NDUFS3
(detects
dNDUFS3)
α-dNDUFS7
α-dNDUFS8
α-dNDUFA5
α-dNDUFA9
α-dNDUFV3
α-dNDUFV1
α-dNDUFV2
α-dNDUFS4
α-dNDUFA7
α-dNDUFA12
α-ATPsynB
*
#

FIGURE S6

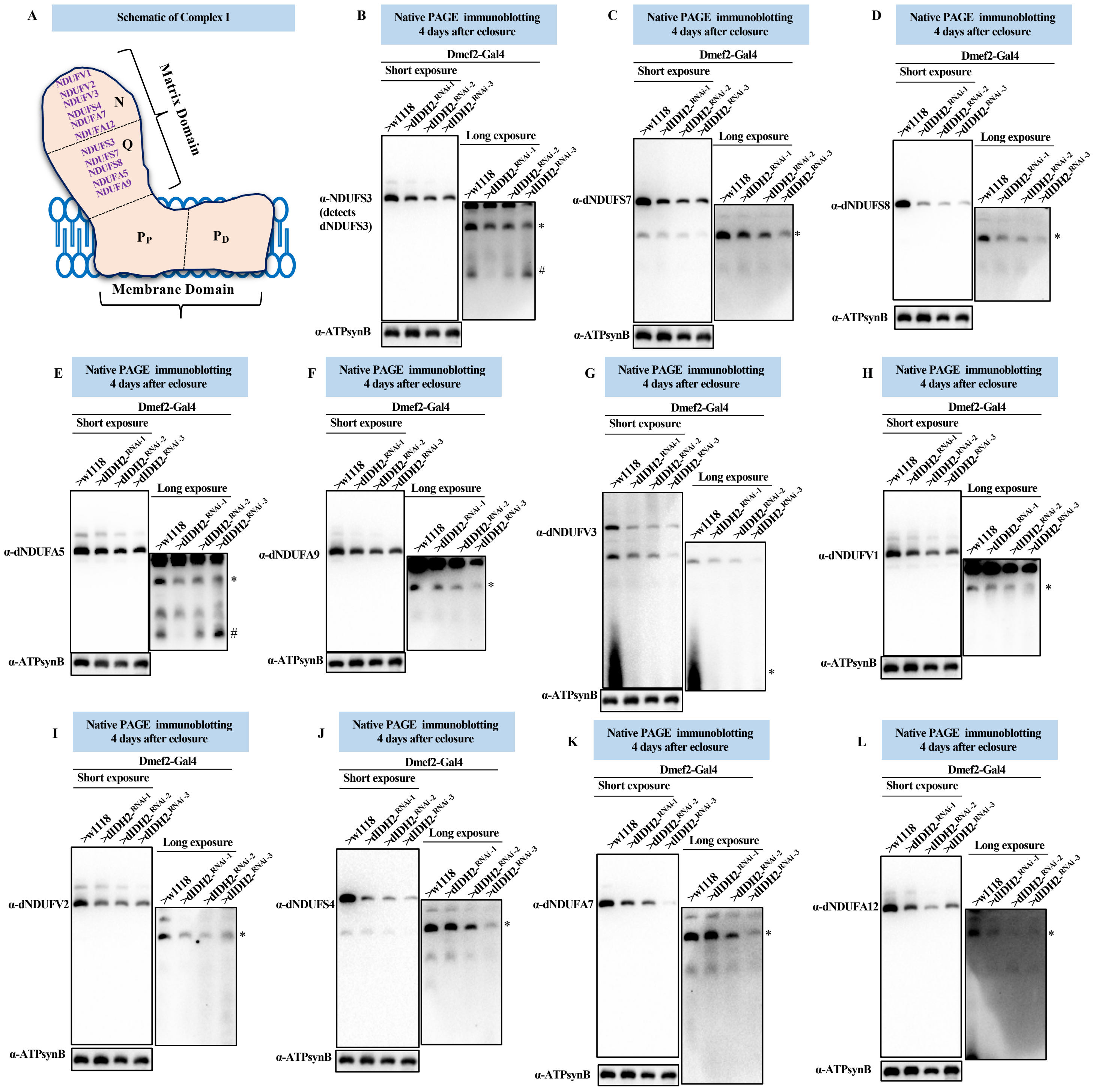

A
Schematic of Complex I
NDUFV1
NDUFV2
NDUFV3
NDUFS4
NDUFA7
NDUFA12
N
NDUFS3
NDUFS7
NDUFS8
NDUFA5
NDUFA9
Q
Matrix Domain
PP
PD
Membrane Domain
B
Native PAGE immunoblotting
4 days after eclosure
Dmef2-Gal4
Short exposure
Long exposure
>w1118
>dIDH2 RNAi-1
>dIDH2 RNAi-2
>dIDH2 RNAi-3
α-NDUFS3 (detects dNDUFS3)
α-ATPsynB
C
α-dNDUFS7
D
α-dNDUFS8
E
α-dNDUFA5
F
α-dNDUFA9
G
α-dNDUFV3
H
α-dNDUFV1
I
α-dNDUFV2
J
α-dNDUFS4
K
α-dNDUFA7
L
α-dNDUFA12

FIGURE S7

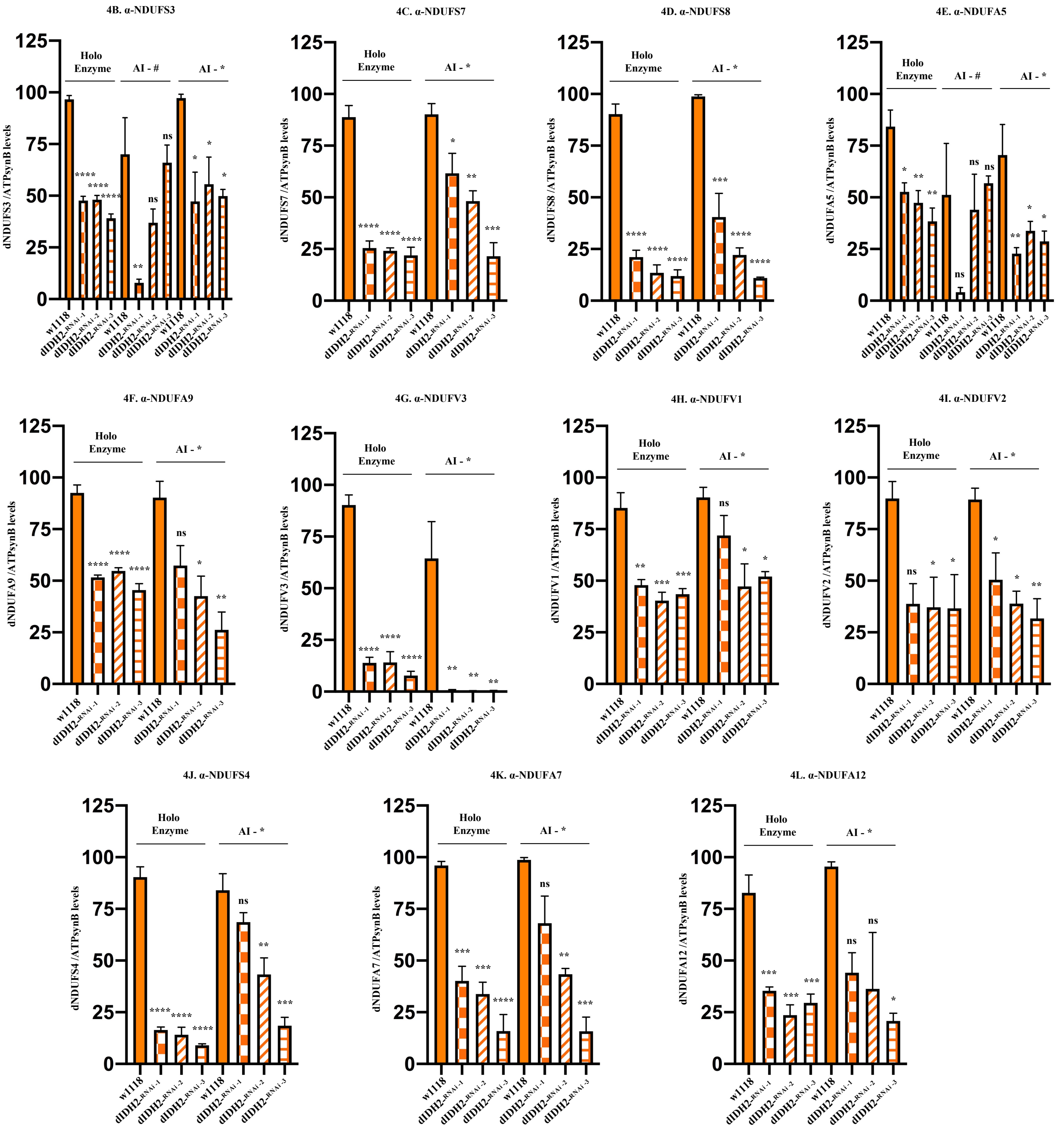

4B. α-NDUFS3
4C. α-NDUFS7
4D. α-NDUFS8
4E. α-NDUFA5
4F. α-NDUFA9
4G. α-NDUFV3
4H. α-NDUFV1
4I. α-NDUFV2
4J. α-NDUFS4
4K. α-NDUFA7
4L. α-NDUFA12
Holo Enzyme
AI - #
AI - *
dNDUFS3 /ATPsynB levels
dNDUFS7 /ATPsynB levels
dNDUFS8 /ATPsynB levels
dNDUFA5 /ATPsynB levels
dNDUFA9 /ATPsynB levels
dNDUFV3 /ATPsynB levels
dNDUFV1 /ATPsynB levels
dNDUFV2 /ATPsynB levels
dNDUFS4 /ATPsynB levels
dNDUFA7 /ATPsynB levels
dNDUFA12 /ATPsynB levels
w1118
dIDH2-RNAi-1
dIDH2-RNAi-2
dIDH2-RNAi-3

FIGURE S8

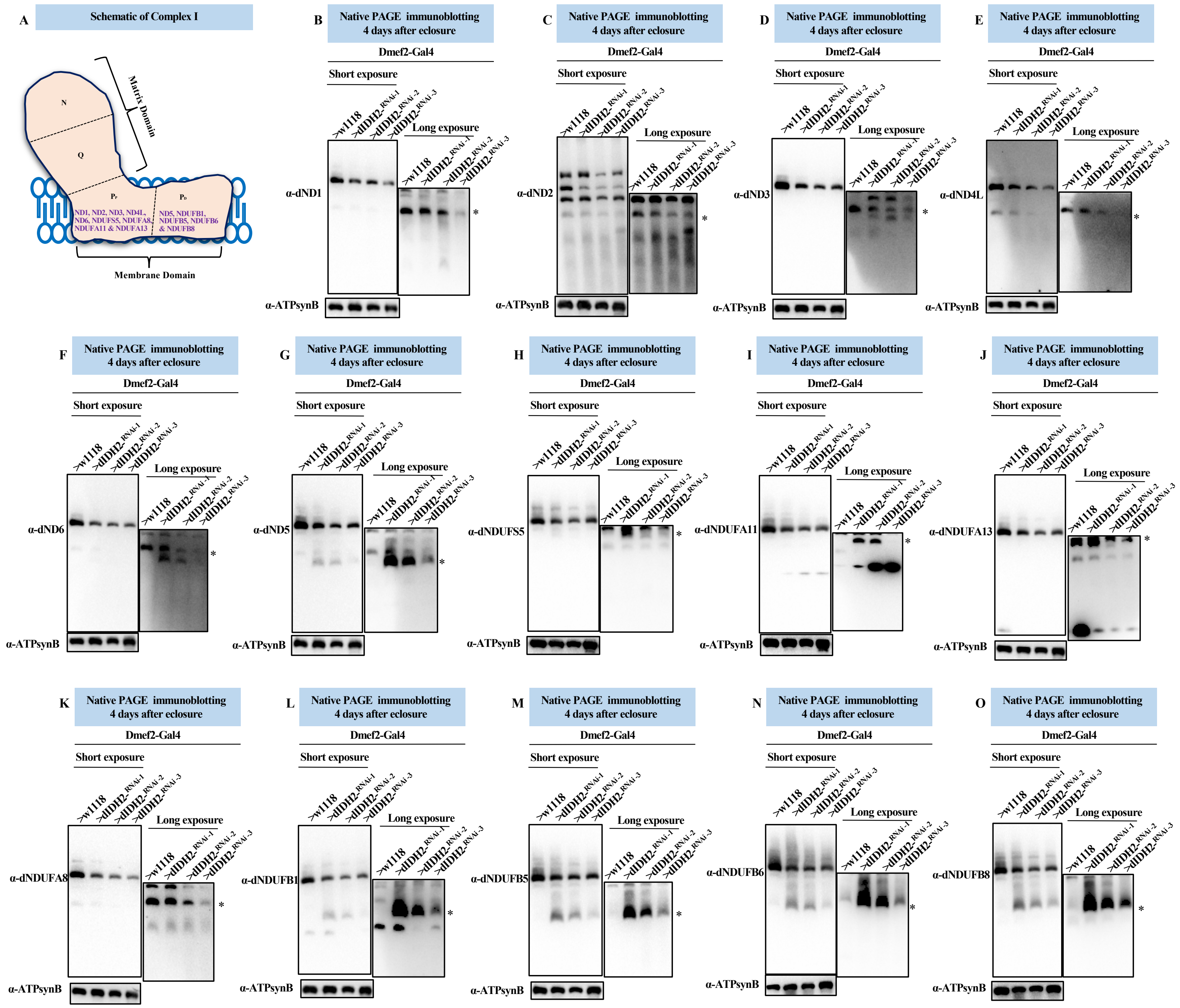
A
Schematic of Complex I
Matrix Domain
N
Q
Pp
PD
ND1, ND2, ND3, ND4L, ND6, NDUFS5, NDUFA8, NDUFA11 & NDUFA13
ND5, NDUFB1, NDUFB5, NDUFB6 & NDUFB8
Membrane Domain
B
C
D
E
F
G
H
I
J
K
L
M
N
O
Native PAGE immunoblotting
4 days after eclosure
Dmef2-Gal4
Short exposure
Long exposure
>w1118
>dIDH2RNAi-1
>dIDH2RNAi-2
>dIDH2RNAi-3
α-dND1
α-dND2
α-dND3
α-dND4L
α-dND6
α-dND5
α-dNDUFS5
α-dNDUFA11
α-dNDUFA13
α-dNDUFA8
α-dNDUFB1
α-dNDUFB5
α-dNDUFB6
α-dNDUFB8
α-ATPsynB
*

FIGURE S9

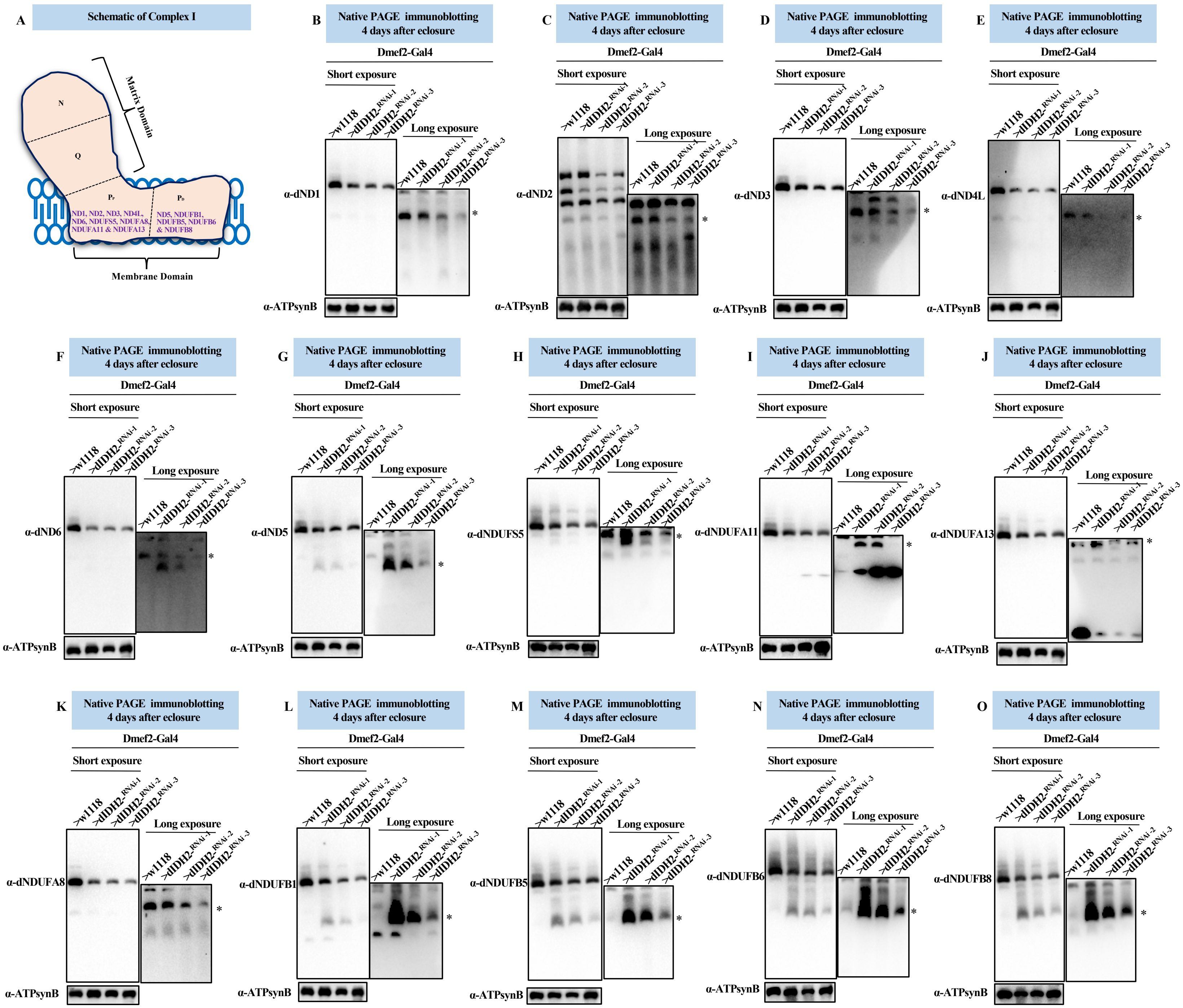
A
Schematic of Complex I
Matrix Domain
N
Q
ND1, ND2, ND3, ND4L, ND6, NDUFS5, NDUFA8, NDUFA11 & NDUFA13
ND5, NDUFB1, NDUFB5, NDUFB6 & NDUFB8
Membrane Domain
B
Native PAGE immunoblotting
4 days after eclosure
Dmef2-Gal4
Short exposure
Long exposure
w1118
dIDH2 RNAi-1
dIDH2 RNAi-2
dIDH2 RNAi-3
α-dND1
α-ATPsynB
C
α-dND2
D
α-dND3
E
α-dND4L
F
α-dND6
G
α-dND5
H
α-dNDUFS5
I
α-dNDUFA11
J
α-dNDUFA13
K
α-dNDUFA8
L
α-dNDUFB1
M
α-dNDUFB5
N
α-dNDUFB6
O
α-dNDUFB8
*

FIGURE S10

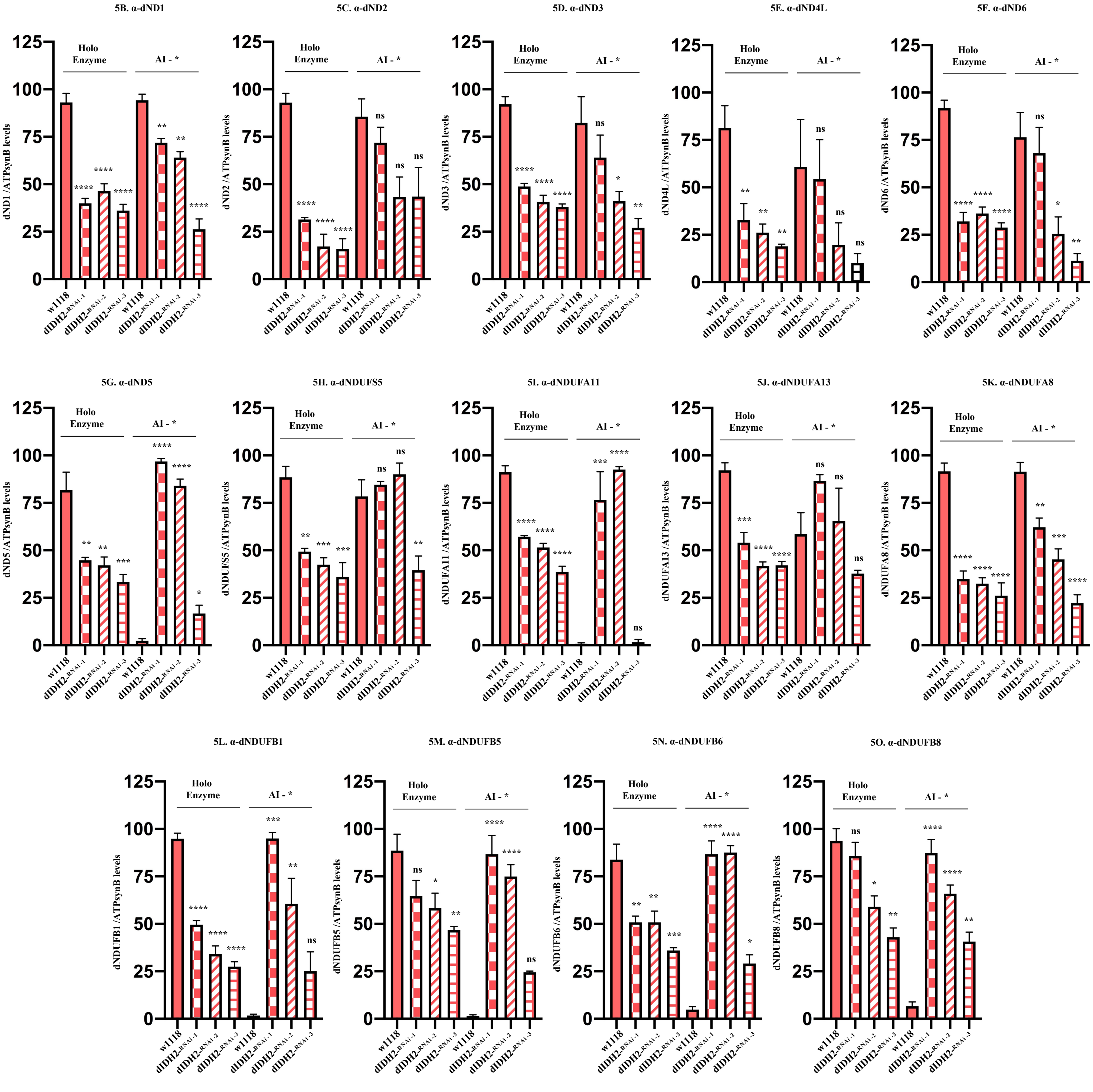

5B. α-dND1
5C. α-dND2
5D. α-dND3
5E. α-dND4L
5F. α-dND6
5G. α-dND5
5H. α-dNDUFS5
5I. α-dNDUFA11
5J. α-dNDUFA13
5K. α-dNDUFA8
5L. α-dNDUFB1
5M. α-dNDUFB5
5N. α-dNDUFB6
5O. α-dNDUFB8
dND1 /ATPsynB levels
dND2 /ATPsynB levels
dND3 /ATPsynB levels
dND4L /ATPsynB levels
dND6 /ATPsynB levels
dND5 /ATPsynB levels
dNDUFS5 /ATPsynB levels
dNDUFA11 /ATPsynB levels
dNDUFA13 /ATPsynB levels
dNDUFA8 /ATPsynB levels
dNDUFB1 /ATPsynB levels
dNDUFB5 /ATPsynB levels
dNDUFB6 /ATPsynB levels
dNDUFB8 /ATPsynB levels
Holo Enzyme
AI - *
0
25
50
75
100
125
w1118
dIDH2-RNAi-1
dIDH2-RNAi-2
dIDH2-RNAi-3

FIGURE S11

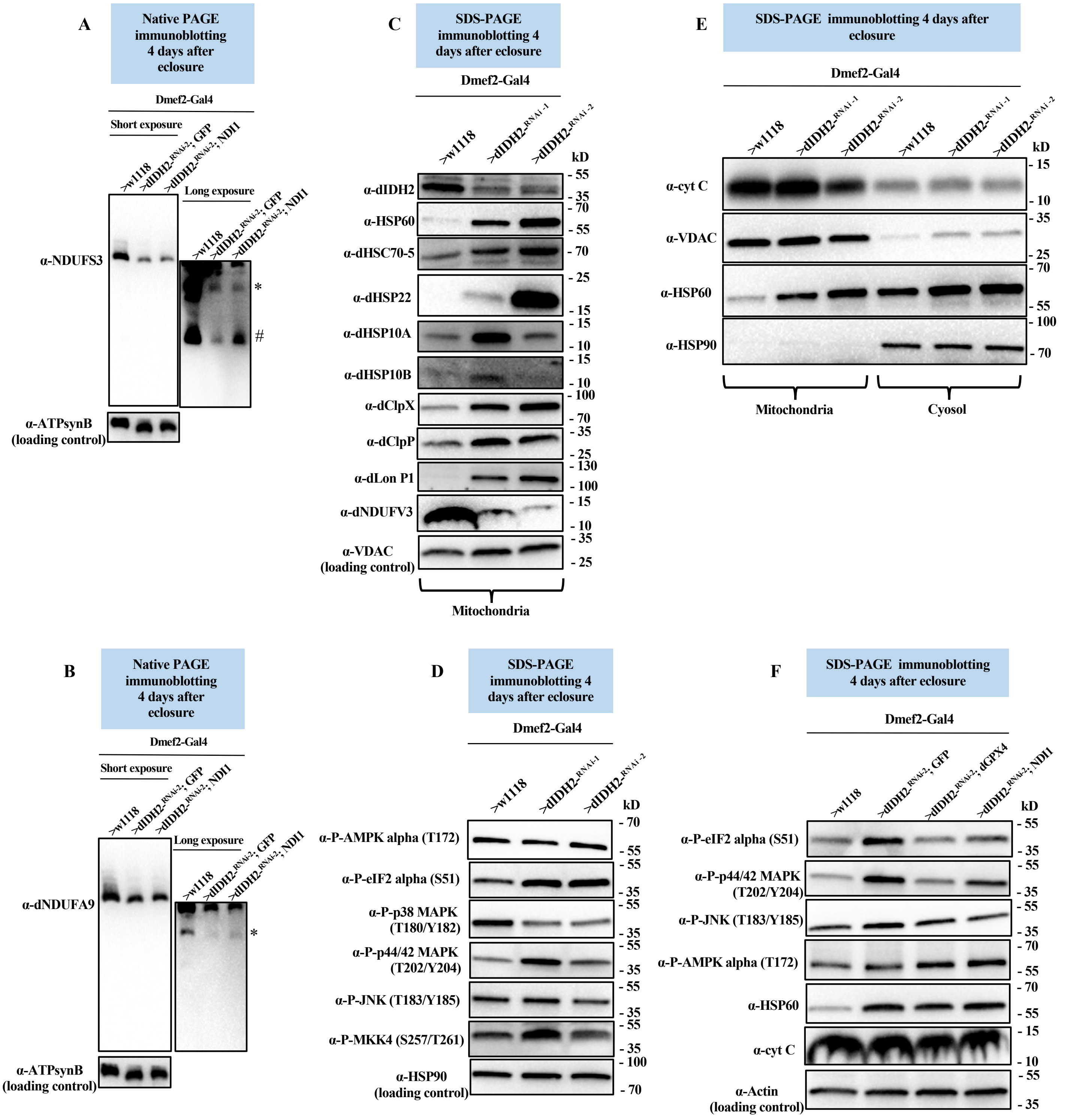
A
Native PAGE immunoblotting 4 days after eclosure
Dmef2-Gal4
Short exposure
>w1118
>dIDH2RNAi-2; GFP
>dIDH2RNAi-2; NDI1
Long exposure
α-NDUFS3
*
#
α-ATPsynB (loading control)
B
Native PAGE immunoblotting 4 days after eclosure
Dmef2-Gal4
Short exposure
Long exposure
α-dNDUFA9
*
α-ATPsynB (loading control)
C
SDS-PAGE immunoblotting 4 days after eclosure
Dmef2-Gal4
>w1118
>dIDH2RNAi-1
>dIDH2RNAi-2
kD
α-dIDH2
α-HSP60
α-dHSC70-5
α-dHSP22
α-dHSP10A
α-dHSP10B
α-dClpX
α-dClpP
α-dLon P1
α-dNDUFV3
α-VDAC (loading control)
Mitochondria
D
SDS-PAGE immunoblotting 4 days after eclosure
Dmef2-Gal4
α-P-AMPK alpha (T172)
α-P-eIF2 alpha (S51)
α-P-p38 MAPK (T180/Y182)
α-P-p44/42 MAPK (T202/Y204)
α-P-JNK (T183/Y185)
α-P-MKK4 (S257/T261)
α-HSP90 (loading control)
E
SDS-PAGE immunoblotting 4 days after eclosure
Dmef2-Gal4
α-cyt C
α-VDAC
α-HSP60
α-HSP90
Mitochondria
Cyosol
F
SDS-PAGE immunoblotting 4 days after eclosure
Dmef2-Gal4
>w1118
>dIDH2RNAi-2, GFP
>dIDH2RNAi-2, dGPX4
>dIDH2RNAi-2, NDI1
α-P-eIF2 alpha (S51)
α-P-p44/42 MAPK (T202/Y204)
α-P-JNK (T183/Y185)
α-P-AMPK alpha (T172)
α-HSP60
α-cyt C
α-Actin (loading control)

FIGURE S12

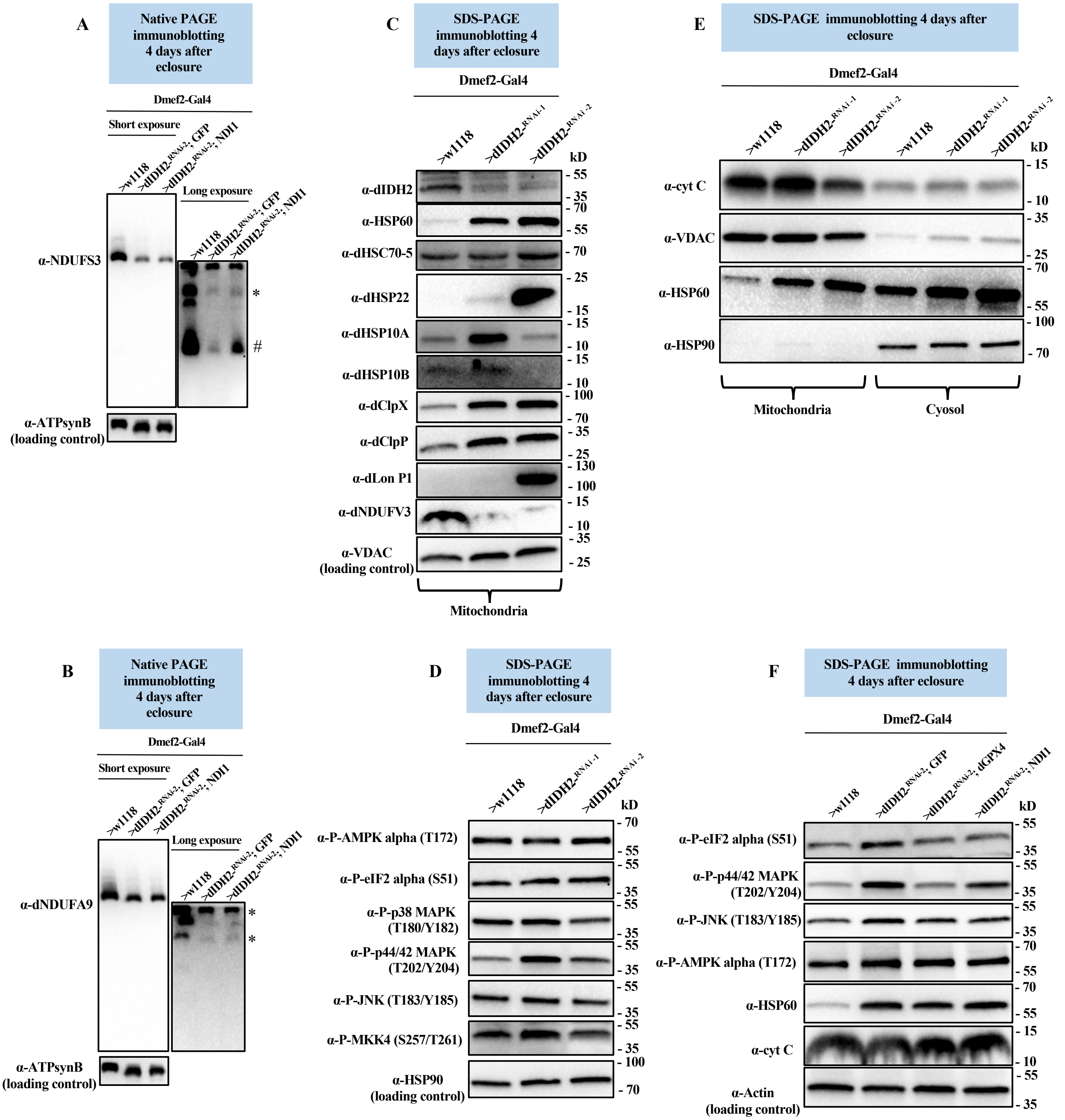
A
Native PAGE immunoblotting 4 days after eclosure
Dmef2-Gal4
Short exposure
>w1118
>dIDH2RNAi-2; GFP
>dIDH2RNAi-2; NDI1
Long exposure
α-NDUFS3
*
#
α-ATPsynB (loading control)
C
SDS-PAGE immunoblotting 4 days after eclosure
Dmef2-Gal4
>w1118
>dIDH2RNAi-1
>dIDH2RNAi-2
kD
α-dIDH2
α-HSP60
α-dHSC70-5
α-dHSP22
α-dHSP10A
α-dHSP10B
α-dClpX
α-dClpP
α-dLon P1
α-dNDUFV3
α-VDAC (loading control)
Mitochondria
E
SDS-PAGE immunoblotting 4 days after eclosure
Dmef2-Gal4
α-cyt C
α-VDAC
α-HSP60
α-HSP90
Mitochondria
Cyosol
B
Native PAGE immunoblotting 4 days after eclosure
α-dNDUFA9
α-ATPsynB (loading control)
D
SDS-PAGE immunoblotting 4 days after eclosure
α-P-AMPK alpha (T172)
α-P-eIF2 alpha (S51)
α-P-p38 MAPK (T180/Y182)
α-P-p44/42 MAPK (T202/Y204)
α-P-JNK (T183/Y185)
α-P-MKK4 (S257/T261)
α-HSP90 (loading control)
F
SDS-PAGE immunoblotting 4 days after eclosure
>dIDH2RNAi-2; dGPX4
α-P-eIF2 alpha (S51)
α-P-p44/42 MAPK (T202/Y204)
α-P-JNK (T183/Y185)
α-P-AMPK alpha (T172)
α-HSP60
α-cyt C
α-Actin (loading control)

FIGURE S13

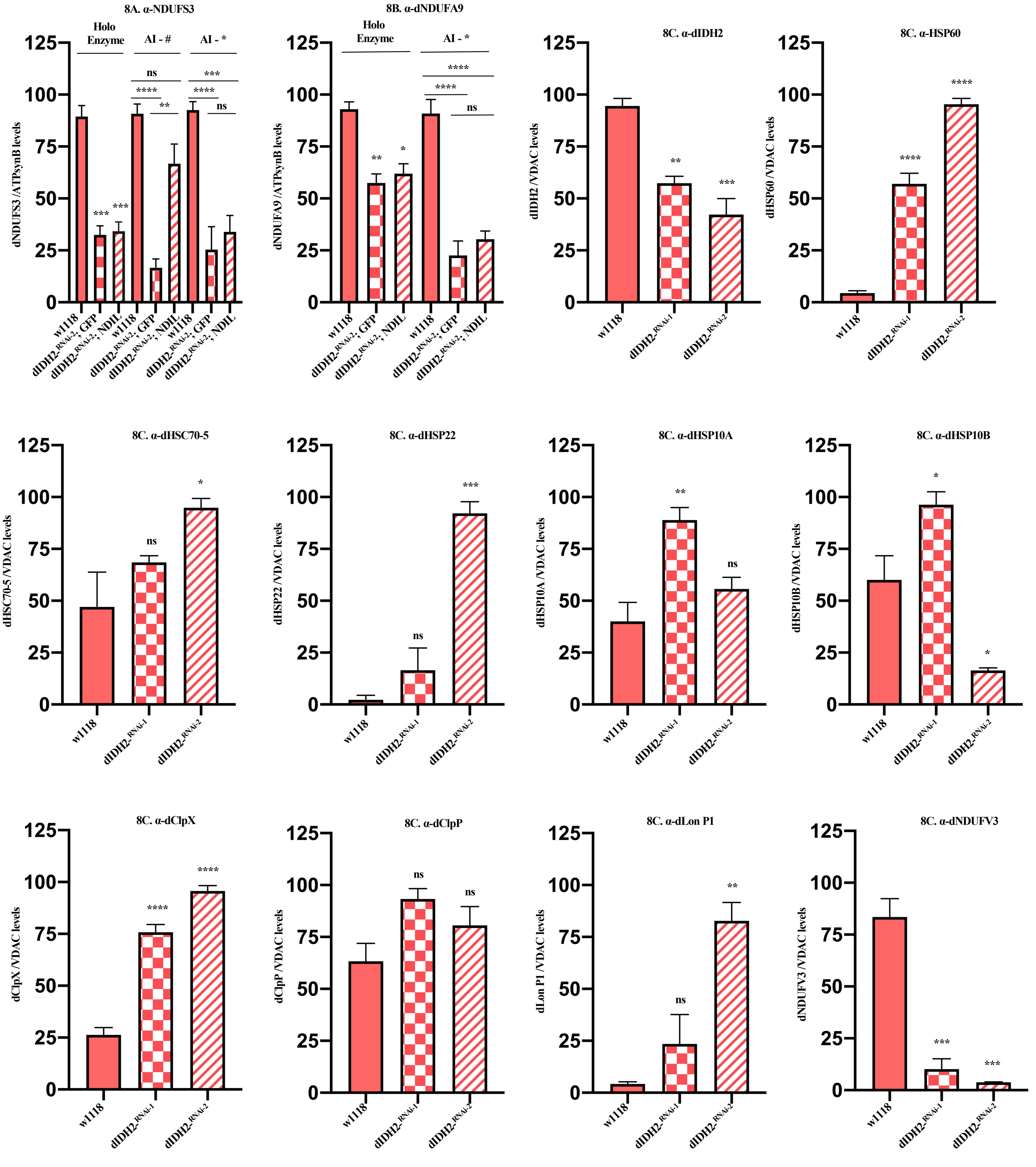
8A. α-NDUFS3
Holo Enzyme
AI - #
AI - *
ns
****
**
***
***
dNDUFS3 /ATPsynB levels
w1118
dIDH2-RNAi-2; GFP
dIDH2-RNAi-2; NDIL
8B. α-dNDUFA9
Holo Enzyme
AI - *
dNDUFA9 /ATPsynB levels
8C. α-dIDH2
dIDH2 /VDAC levels
8C. α-HSP60
dHSP60 /VDAC levels
dIDH2-RNAi-1
dIDH2-RNAi-2
8C. α-dHSC70-5
dHSC70-5 /VDAC levels
8C. α-dHSP22
dHSP22 /VDAC levels
8C. α-dHSP10A
dHSP10A /VDAC levels
8C. α-dHSP10B
dHSP10B /VDAC levels
8C. α-dClpX
dClpX /VDAC levels
8C. α-dClpP
dClpP /VDAC levels
8C. α-dLon P1
dLon P1 /VDAC levels
8C. α-dNDUFV3
dNDUFV3 /VDAC levels
0
25
50
75
100
125

FIGURE S14

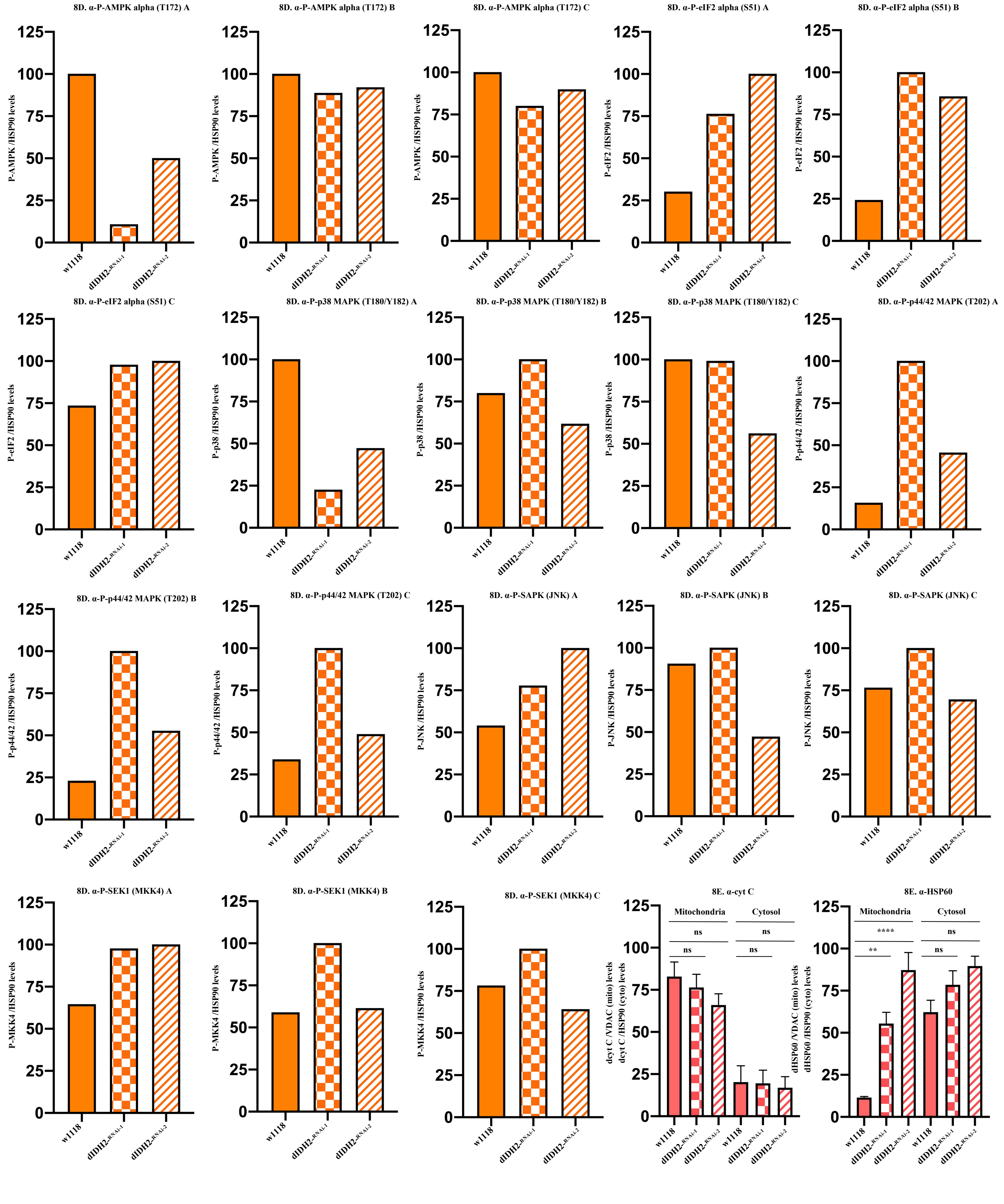
8D. α-P-AMPK alpha (T172) A
8D. α-P-AMPK alpha (T172) B
8D. α-P-AMPK alpha (T172) C
8D. α-P-eIF2 alpha (S51) A
8D. α-P-eIF2 alpha (S51) B
8D. α-P-eIF2 alpha (S51) C
8D. α-P-p38 MAPK (T180/Y182) A
8D. α-P-p38 MAPK (T180/Y182) B
8D. α-P-p38 MAPK (T180/Y182) C
8D. α-P-p44/42 MAPK (T202) A
8D. α-P-p44/42 MAPK (T202) B
8D. α-P-p44/42 MAPK (T202) C
8D. α-P-SAPK (JNK) A
8D. α-P-SAPK (JNK) B
8D. α-P-SAPK (JNK) C
8D. α-P-SEK1 (MKK4) A
8D. α-P-SEK1 (MKK4) B
8D. α-P-SEK1 (MKK4) C
8E. α-cyt C
8E. α-HSP60
P-AMPK /HSP90 levels
P-eIF2 /HSP90 levels
P-p38 /HSP90 levels
P-p44/42 /HSP90 levels
P-JNK /HSP90 levels
P-MKK4 /HSP90 levels
dcyt C /VDAC (mito) levels
dcyt C /HSP90 (cyto) levels
dHSP60 /VDAC (mito) levels
dHSP60 /HSP90 (cyto) levels
Mitochondria
Cytosol
ns
**
****
0
25
50
75
100
125
w1118
dIDH2 RNAi-1
dIDH2 RNAi-2

FIGURE S15

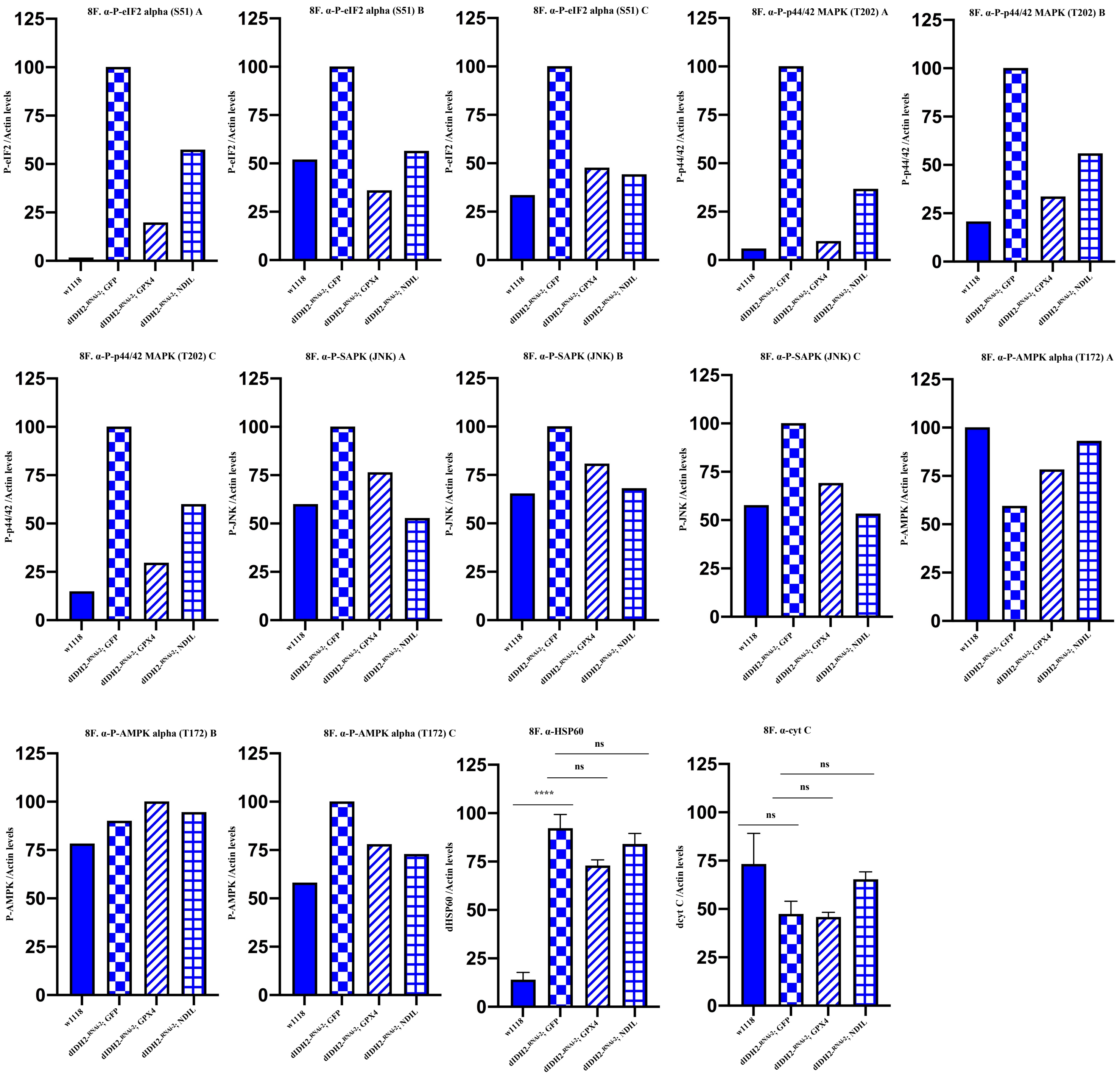
8F. α-P-eIF2 alpha (S51) A
8F. α-P-eIF2 alpha (S51) B
8F. α-P-eIF2 alpha (S51) C
P-eIF2 /Actin levels
8F. α-P-p44/42 MAPK (T202) A
8F. α-P-p44/42 MAPK (T202) B
8F. α-P-p44/42 MAPK (T202) C
P-p44/42 /Actin levels
8F. α-P-SAPK (JNK) A
8F. α-P-SAPK (JNK) B
8F. α-P-SAPK (JNK) C
P-JNK /Actin levels
8F. α-P-AMPK alpha (T172) A
8F. α-P-AMPK alpha (T172) B
8F. α-P-AMPK alpha (T172) C
P-AMPK /Actin levels
8F. α-HSP60
dHSP60 /Actin levels
8F. α-cyt C
dcyt C /Actin levels
0
25
50
75
100
125
w1118
dIDH2 RNAi-2; GFP
dIDH2 RNAi-2; GPX4
dIDH2 RNAi-2; NDIL
****
ns

FIGURE S16

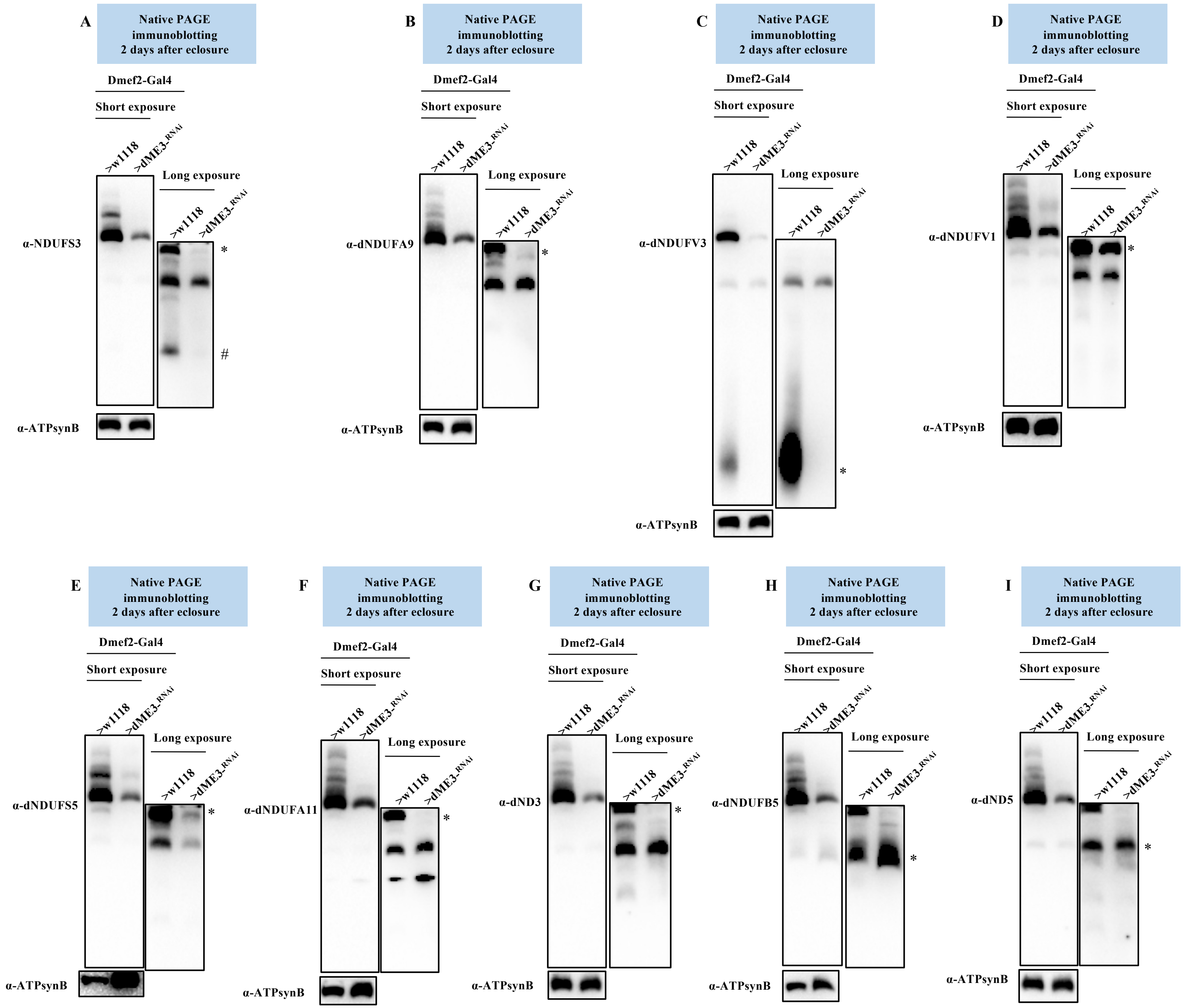
A
Native PAGE immunoblotting 2 days after eclosure
Dmef2-Gal4
Short exposure
>w1118
>dME3-RNAi
Long exposure
α-NDUFS3
*
#
α-ATPsynB
B
α-dNDUFA9
C
α-dNDUFV3
D
α-dNDUFV1
E
α-dNDUFS5
F
α-dNDUFA11
G
α-dND3
H
α-dNDUFB5
I
α-dND5

FIGURE S17

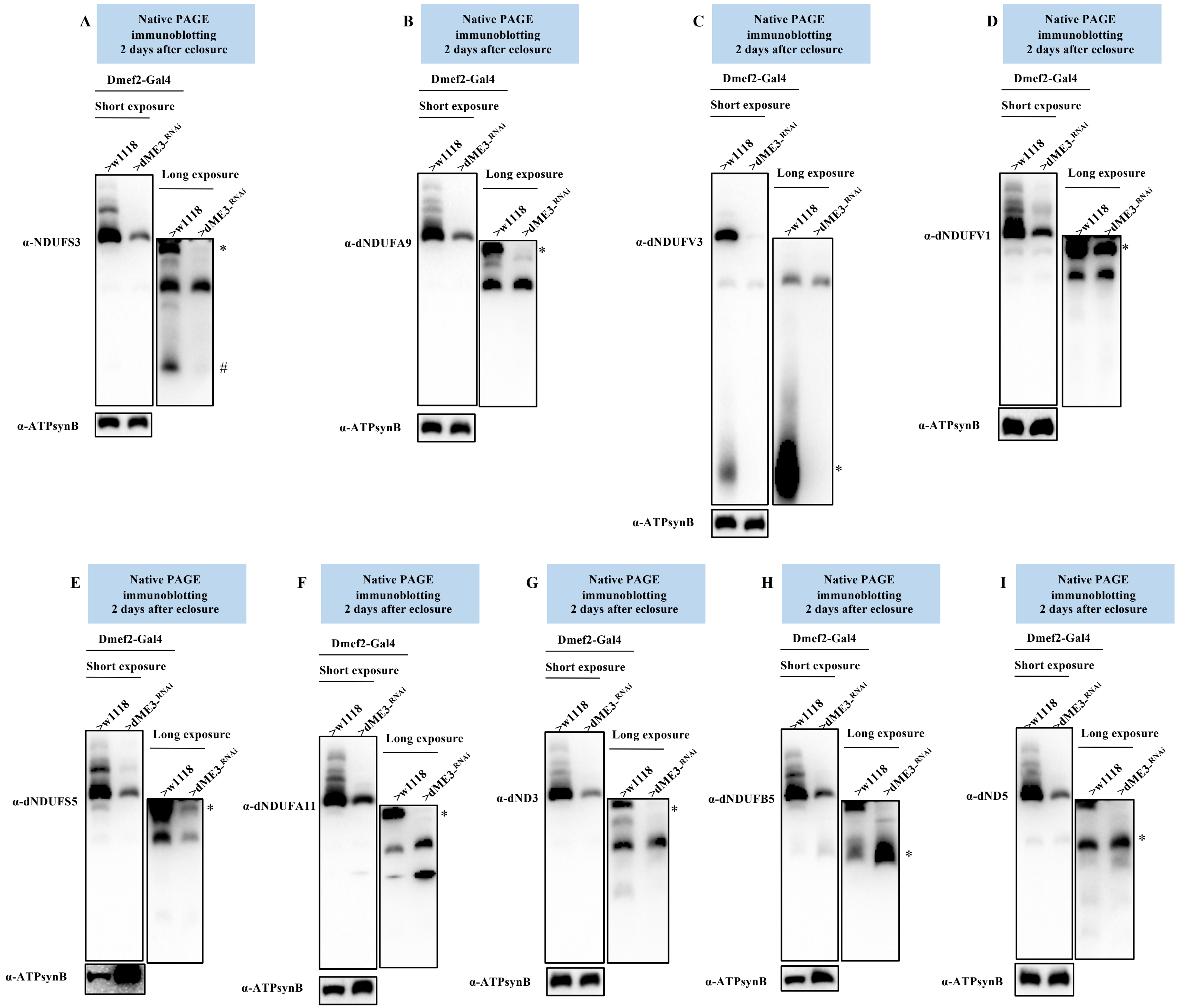
A
Native PAGE immunoblotting 2 days after eclosure
Dmef2-Gal4
Short exposure
>w1118
>dME3-RNAi
Long exposure
α-NDUFS3
*
#
α-ATPsynB
B
α-dNDUFA9
C
α-dNDUFV3
D
α-dNDUFV1
E
α-dNDUFS5
F
α-dNDUFA11
G
α-dND3
H
α-dNDUFB5
I
α-dND5

FIGURE S18

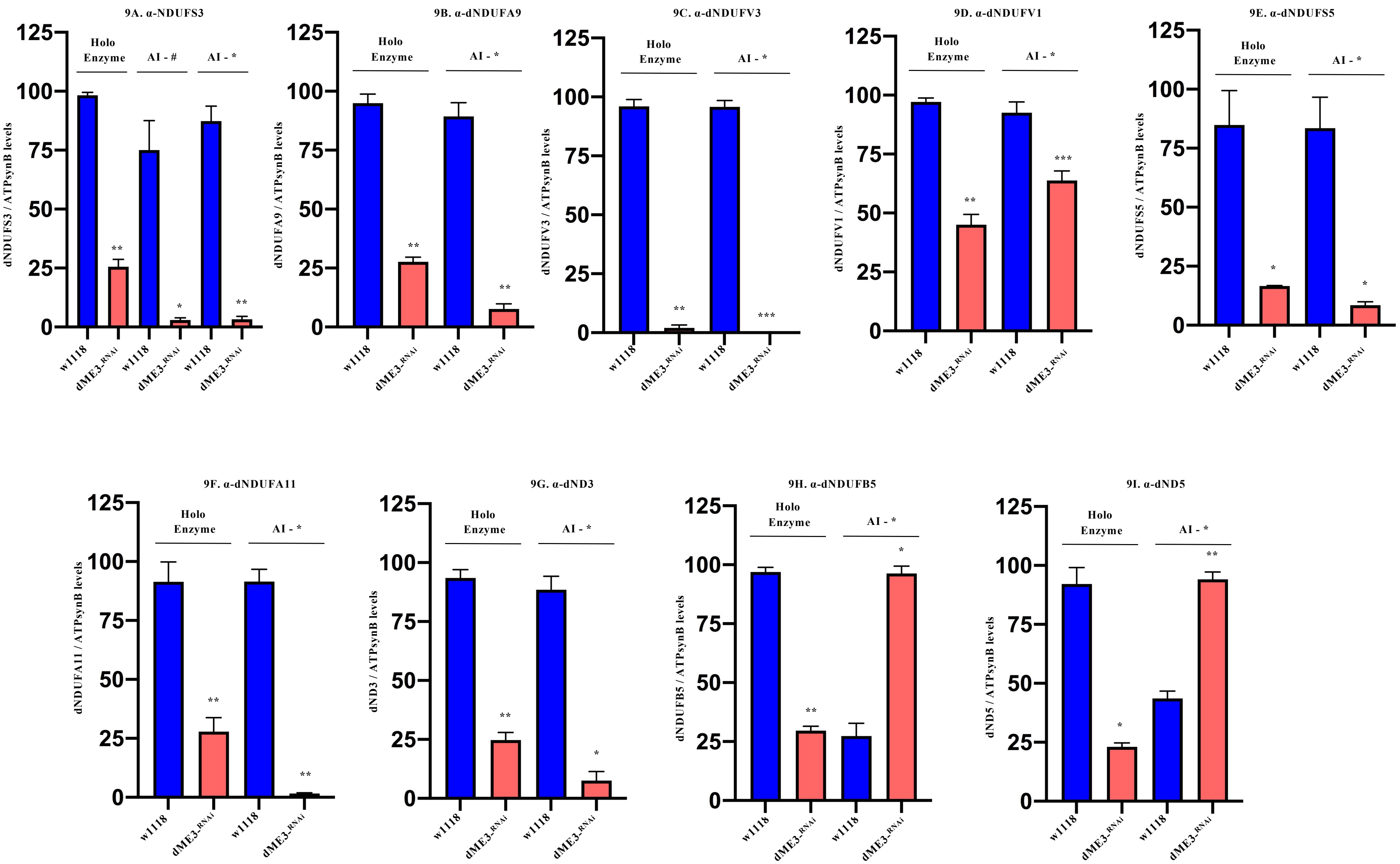

9A. α-NDUFS3
dNDUFS3 / ATPsynB levels
Holo Enzyme
AI - #
AI - *
9B. α-dNDUFA9
dNDUFA9 / ATPsynB levels
9C. α-dNDUFV3
dNDUFV3 / ATPsynB levels
9D. α-dNDUFV1
dNDUFV1 / ATPsynB levels
9E. α-dNDUFS5
dNDUFS5 / ATPsynB levels
9F. α-dNDUFA11
dNDUFA11 / ATPsynB levels
9G. α-dND3
dND3 / ATPsynB levels
9H. α-dNDUFB5
dNDUFB5 / ATPsynB levels
9I. α-dND5
dND5 / ATPsynB levels
w1118
dME3-RNAi
0
25
50
75
100
125
