# Supplementary material for: IDH2-mediated regulation of the biogenesis of the oxidative phosphorylation system
Source: Sci Adv. 2022 May 11;8(19):eabl8716. doi: 10.1126/sciadv.abl8716 (PMC9094667; doi:10.1126/sciadv.abl8716)
Supplement: Supplementary file 2 — Tables S1 to S8 [file sciadv.abl8716_tables_s1_to_s8.zip › sciadv.abl8716_table_s8.docx]

| Peptide Antigen | Target Protein |
| --- | --- |
| SDNLFVHRDTPEDNPN-Cys | dNDUFV2 (CG5703) |
| Cys-GINFAWNKRTRVSTK | dNDUFS4 (CG12203) |
| Cys-KQWDWPPAQIMEPKV | dNDUFA5 (CG6463) |
| EDGLADRTQPQPEIPDGPSH-Cys | dNDUFA7 (CG3621) |
| Cys-DKSALYGRPAGSEGKAPSW | dNDUFB1 (CG18624) |
| SPPKCIEARDELRLFA | dNDUFA9 (CG6020) |
| GGYKKIPFARVPPKS-Cys | dNDUFA13 (CG3446) |
| Cys-GNINAVTRRDYQET | dIDH2 (CG7176) |
| Cys-DTSGEAKKEEKN | Hsc70-5 (CG8542) |
| Cys-KKSAEEPNDKAASQ | Hsp22 (CG4460) |
| GTRNASTGNHIPIG-Cys | dHsp10A (CG11267) |
| GARNPAGAGHLSVG-Cys | dHsp10B (CG9920) |
| AKGSGAGQTHGSARC | dClpX (CG4538) |
| EQTGRGERAYDIFSR-Cys | dClpP (CG5045) |
| Cys-AKSETWPYS | dLon P1 (CG8798) |

**Table S8: A list of the peptide antigens and their target proteins.**
